# A redescription of *Glyptochelone suyckerbuykii* (Ubaghs, 1879), an enigmatic fossil sea turtle (*Chelonioidea*) from the Maastrichtian of the Netherlands and Belgium, sheds new light on fossil sea turtle shell variation and neural bone homology

**DOI:** 10.1186/s13358-025-00389-y

**Published:** 2025-10-01

**Authors:** Juliette C. L. Menon, Walter G. Joyce

**Affiliations:** Department of Geosciences, University of Fribourg, Chemin du Musée 6, 1700 Fribourg, Switzerland

**Keywords:** *Pan-Chelonioidea*, Sea turtles, µCT, Neural series, Thoracic vertebrae, Ornamentation, Shell surface texture, Homology

## Abstract

We here provide a redescription the Late Cretaceous (Campanian–Maastrichtian) fossil marine turtle *Glyptochelone suyckerbuykii* to document its anatomy and intraspecific variation. This redescription highlights the complete absence of a nuchal pedestal, presence of a radiating shell surface texture pattern easily differentiated from that of coeval marine turtles, and the presence of interneural elements, a characteristic unique among Late Cretaceous turtles. A phylogenetic analysis suggests that *Glyptochelone suyckerbuykii* is located at the base of *Dermochelyidae*, a dubious result likely resulting from missing data. To allow constructing phylogenetic characters based on the presence of supernumerary neural elements, we suggest a revised classification for the midline column of the carapace of turtles based on novel homology criteria as consisting of neurals, which are outgrowths of the neural arches, as opposed to preneurals, interneurals, and postneurals, which are independent bones that form in front, within, and posterior to the neural column, respectively. We suggest use of this novel classification in future phylogenetic analysis.

**Supplementary Information:**

The online version contains supplementary material available at 10.1186/s13358-025-00389-y.

## Introduction

The clade of extant sea turtles (*Chelonioidea*) is a fascinating group of marine reptiles that includes two extant lineages, the monospecific leatherback turtle (*Dermochelys coriacea*) and the hard-shelled *Cheloniidae* (Joyce et al., 2021a). Chelonioids display numerous skeletal adaptations to the marine realm, such as flippers (e.g., Deraniyagala, 1939; Krahl, 2021; Seago, 1979), porous bones (e.g., Clarac et al., 2020; Scheyer & Sander, 2007; Scheyer et al., 2014), and the loss of neck retraction (e.g., Hermanson et al., 2022; Jones et al., 2012). The group displays important variation to the degree of ossification of the shell (e.g., Deraniyagala, 1939; Hirayama, 1997; Kordikova, 2002) and, notably for this contribution, the composition of the neural series, which fully disappeared in the lineage leading up to the extant leatherback turtle, *Dermochelys coriacea* (e.g., Deraniyagala, 1939; Gervais, 1872; Seago, 1979; Völker, 1913), but proliferated in some extant hard-shelled cheloniids, including *Lepidochelys olivacea*, resulting in the presence of additional midline plates (e.g., Kordikova, 2002; Misuri, 1910; Pritchard, 1988; Zangerl & Turnbull, 1955).

The relationships between the additional midline plates (herein termed preneurals, interneurals, and postneurals based on their position within the neural series, see below) and the thoracic neural arches in pan-cheloniids were first investigated by Zangerl and Turnbull (1955) who described the visceral face of the shell of *Procolpochelys grandaeva*, a Miocene fossil cheloniid from the New Jersey (USA) with an unusually high number of midline elements. Zangerl and Turnbull (1955) noticed that the neural series of turtles displaying supernumerary midline plates is formed by an alternation of smaller elements confluent with the neural arches and larger elements devoid of a contact with a neural arch, but they did not further investigate the utility of their observations regarding the homology of neural elements, perhaps because the interconnectivity of midline plates with the underlying neural arches cannot be assessed in most specimens. Supernumerary elements have otherwise been reported from the front or back of the neural series of other turtles [e.g., the “preneurals” of baenids (Gilmore, 1919), sinemydids (Brinkman & Peng, 1993), and trionychids (Joyce, 2025a), or the “postneurals” or “intermediate elements” of cheloniids (Weems, 1974), nanhsiungchelyids (Riggs, 1906), and plesiochelyids (Anquetin et al., 2014)] and from within the neural series [e.g., the elevated epineurals of *ctenochelyids* (Wieland, 1905)], but the homology of these elements remains mostly unclear.

**Fig. 1:**
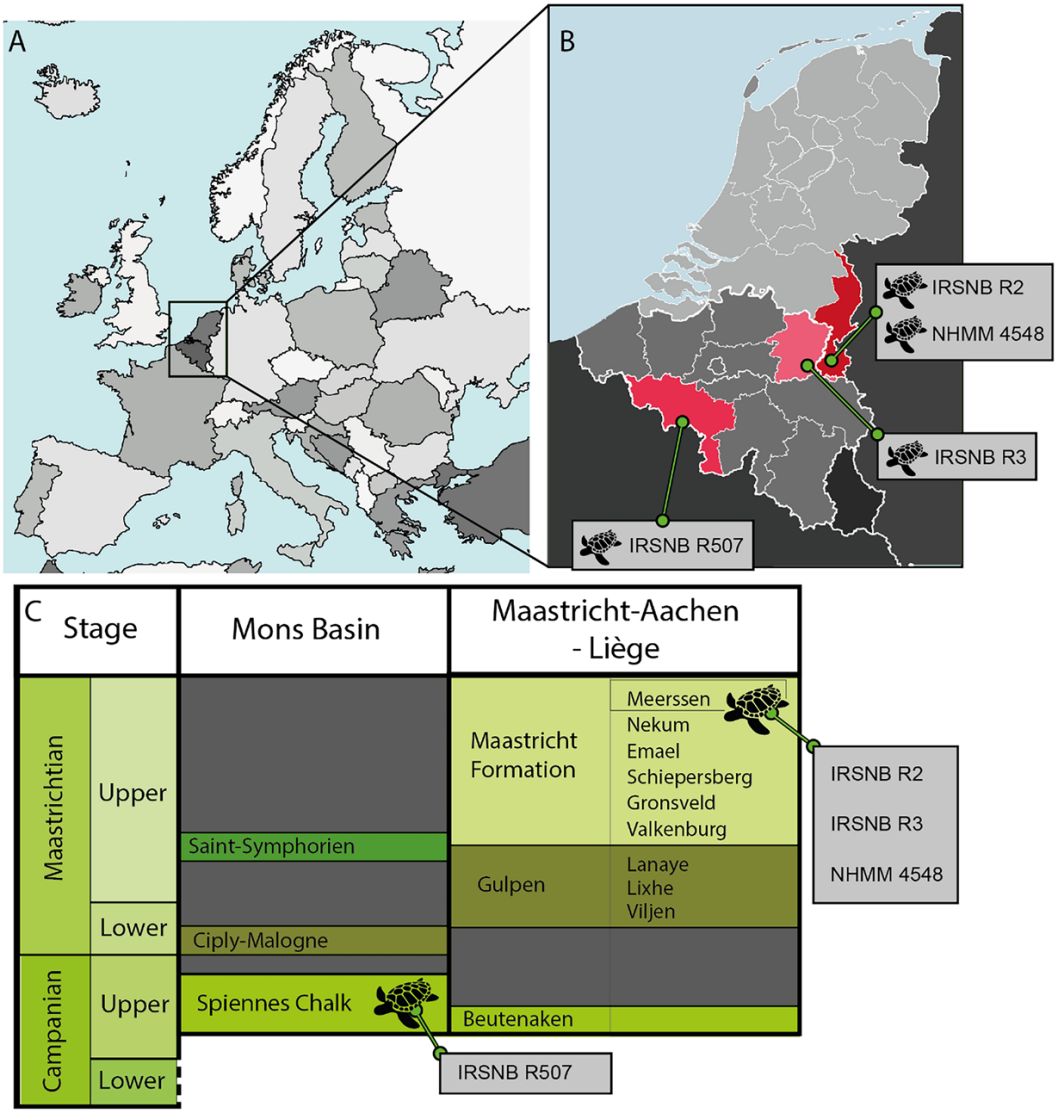
Geographic and stratigraphic repartition of *Glyptochelone suyckerbuykii* occurrences. **A** Map of western Europe; **B** Close-up on the Belgium and the Netherlands showing the localities of the described material of *Glyptochelone suyckerbuykii*; **C** Schematic stratigraphy of Mons Basin and Maastricht area showing the stratigraphic positions of *Glyptochelone suyckerbuykii occurrences* (modified from Keutgen, 2011)

*Glyptochelone suyckerbuykii* was initially published under the binomen *Chelonia suyckerbuykii* Ubaghs, 1879 based on two specimens from the late Maastrichtian (latest Cretaceous) of the Netherlands and Belgium (Fig. 1). The holotype (IRSNB R2) consists of a fairly complete shell that was excavated in 1877 from a late Maastrichtian quarry near the village of Vilt, the Netherlands (Ubaghs, 1879), while the referred specimen (IRSNB R3) is an isolated neural element that was discovered in 1875 in equivalent sediments exposed near the nearby village of Kanne, Belgium (Felder, 1980). Among Late Cretaceous marine turtles, the holotype of *Glyptochelone suyckerbuykii* stands out by its unique shell surface texture and, excluding the nuchal and pygal, the presence of 13 midline plates (Pritchard, 1988; Zangerl, 1960, 1969). In 1899, a third, smaller shell (IRSNB R507) was collected from the late Campanian of Spiennes, Belgium (Mini-Van de Geijn, 1955), but this specimen essentially remains undescribed. As this Late Cretaceous turtle apparently has no close relationships with the extant *Chelonia mydas*, Dollo (1909) erected the new combination *Glyptochelone suyckerbuykii* for this taxon. A fourth specimen (NHMM 4548), which consists of a fairly complete plastron and carapace fragments from the latest Maastrichtian of the Netherlands, was briefly reported by Kruytzer in 1955.

This was followed by the mentioning of two hyoplastral fragments by Hofker (1955), probably from the same horizon, but these now appear to be lost. The taxon *Glyptochelone suyckerbuykii* is regularly listed as valid (e. g Bardet et al., 1996; Dollo, 1909; Felder, 1980; Heere et al., 2023; Hirayama, 1997; Kuhn, 1964; Mulder, 2003, 2012; Zangerl, 1960, 1980), but a detailed reevaluation is outstanding beyond new illustrations (Lapparent de Broin et al., 2018; Nolis et al., 2018), updated data about the geological setting of the material (Felder, 1980; Mulder & Jagt, 2016; Mulder et al., 1998), or additional information about the historical context of the discovery (Mini-Van de Geijn, 1955; Felder, 1980). *Glyptochelone suyckerbuykii* has never been included in a phylogenetic analysis.

We here provide a redescription of *Glyptochelone suyckerbuykii* based on the more complete still available material in order to gain new insights into the morphology of this enigmatic taxon. We hereby focus on documenting intraspecific variability, shell surface texture, and the phylogenetic relationships of this taxon. We furthermore take the occasion to discuss the homology of midline carapacial elements and to propose a new nomenclature for these elements.

## Material and methods

### CT-scan data acquisition

To document the relationship of the thoracic vertebrae to the neurals in an extant cheloniid, we obtained the CT scan data of SMF 63250 *Chelonia mydas* from Morphosource (https://www.morphosource.org/concern/media/000655871). This specimen was subjected to high-resolution micro-computed tomography (µCT) scanning in (2022), using a Nikon HT X 320, at the 3D Imaging Lab—3DImg of the Department of Geosciences of Eberhard Karls Universität, Tübingen, Germany. The scan acquisition was performed using a beam energy of 180 kV, a current of 175 µA, and with an exposure time of 67 ms and an isotropic voxel size of 0.1221408 mm. The scan was reoriented using the re-slice tool of Mimics v. 24 (http://biomedical.materialise.com/mimics) to align the vertebral column with the principal planes of the 3D coordinate system and then manually segmented with the lasso tool. The resulting 3D models were exported as.ply files. All digital renderings used in the figures were performed using Blender v. 3.31 (blender.org). The newly obtained 3D models are deposited in association with the CT scan data at MorphoSource (https://www.morphosource.org/concern/biological_specimens/000655868).

### Phylogenetic analysis

#### Character-taxon matrix

To explore the phylogenetic relationships of *Glyptochelone suyckerbuykii*, we slightly modified the matrix of Menon et al. (2024), which consists of 356 characters for 97 taxa. We chose this matrix because it is the most recent update of the dataset provided by Evers and Benson (2019), a global turtle matrix with an emphasis on marine-adapted turtle groups, including unambiguous pan-chelonioids, protostegids, sandownids, and unambiguous thalassochelydians.

Following our novel assessment of neural bone homology (see Discussion), we modified character 205 (character 204 of Menon et al., 2024) to exclude interneural elements from the neural count (see Additional file 1), which, in return, demanded modifying the scorings for the following taxa: *Lepidochelys kempii* (1, not 0), *Lepidochelys olivacea* (1, not 0), *Procolpochelys charlestonensis* (1, not 0), *Argillochelys cuneiceps* (1, not?), and *Xinjiangchelys radiplicatoides* (1, not?). The matrix was expanded through the addition of *Glyptochelone suyckerbuykii*, but the dubious taxon *Corsochelys haliniches* was omitted following the recommendations of Menon et al. (2024). We finally added three new characters to the matrix.

- Character 201: Presence of a preneural plate covering the first thoracic vertebrae: 0 = absent; 1 = present in some specimens as an intraspecific variable character; 2 = present in all specimens.
- Character 206: Presence of interneural elements within the neural series: 0 = absent; 1 = present in some specimens as an intraspecific variable character; 2 = present in all specimens. This character is scored as inapplicable if the neurals are absent (ch 203.1). This character concerns only symmetrical elements and therefore excludes pathological, intercalary elements.
- Character 207: Maximal total number of interneurals: 0 = one; 1 = two; 2 = more than two. This character is scored as inapplicable if the interneurals are absent (ch 206.0) (see Additional file 1 for complete list of character).

The new matrix consists of 359 characters for 97 taxa (see Additional file 2 matrix in nexus format).

#### Parsimony analysis

Our parsimony analysis was performed with TNT version 1.6 (update from 12 October 2023; Goloboff & Morales, 2023). As in previous studies using our matrix (Evers et al., 2019; Joyce et al., 2021a; Menon et al., 2024), *Proganochelys quenstedtii* was chosen as the outgroup and a backbone constraint of all extant taxa was enforced following the most recent molecular topology (Thomson et al., 2021). The analysis was performed using a New Technology Search with default settings, with tree drifting (Goloboff, 1999) and parsimony ratchet (Nixon, 1999) algorithms enabled. The initial level of driven search and the number of times the minimal tree length should be found to were set to 30. As in previous studies (Evers et al., 2019; Joyce et al., 2021a; Menon et al., 2024), further rounds of tree bisection and reconnection (TBR) to the most parsimonious trees (MPTs) previously obtained were performed. As was done by Menon et al. (2024), we ordered multistate character that form morphoclines (see Joyce, 2025a; Slowinski, 1993; Wilkinson, 1992), including the new character 207. The full list of ordered character is: 7, 15, 19, 22, 35, 62, 66, 68, 75, 77, 78, 80, 91, 95, 96, 105, 109, 119, 125, 132, 133, 140, 144, 149, 207, 210, 225, 256, 291, 302, 315, 335, 346, 347 (using regular numbering starting at 1).

We performed an exploratory analysis with equal weighted characters. The resulting trees are available in Additional file 3.

Taking into account the strong levels of homoplasy generally observed in turtle matrices, including this one, we decided to use implied weighting strategies, as was done by Menon et al. (2024). An initial analysis with a concavity constant of k = 12 was performed following the recommendations of Goloboff et al., 2018. The trees obtained from this analysis are provided in Additional file 4. We also performed an analysis with a stronger concavity constant (k = 7), following the recommendations of Ezcurra (2024). Trees obtained from this analysis are provided in Additional file 5 and their topology is discussed below (see Phylogeny). However, as the analyses using equal weights and k = 7 provided high implausible placements for sandownids (see Phylogeny section below), we only figure and discuss the analysis with a weighting factor of k = 12.

#### Character optimization

To explore character transitions across the topology obtained, we used PAUP* v.4 (Swofford, 1998) which, in contrast to TNT, allows manually choosing the optimality criterion (i.e., ACCTRAN and DELTRAN) to record ambiguous synapomorphies that occur in Case of incomplete data and character conflict between sister taxa. Because character optimization must be performed on a fully resolved tree, we selected a random MPT of the above-mentioned TNT analyses. The chosen trees, number 2 and number 3, as well as the complete list of synapomorphies for both analyses with implied weighting (k = 12 and k = 7) are available in the Additional files 6 and 7, respectively.

## Systematic paleontology

*Testudinata* Klein, 1760 (sensu Joyce et al., 2020).

*Pan-Chelonioidea* Joyce et al., 2004 (sensu Joyce et al., 2021b).

*Glyptochelone suyckerbuykii* (Ubaghs, 1879).

**Taxonomic history.** *Chelonia suyckerbuykii* Ubaghs, 1879 (new species); *Glyptochelone suyckerbuyki* Dollo, 1909 (new combination, misspelling of the species epithet).

**Fig. 2:**
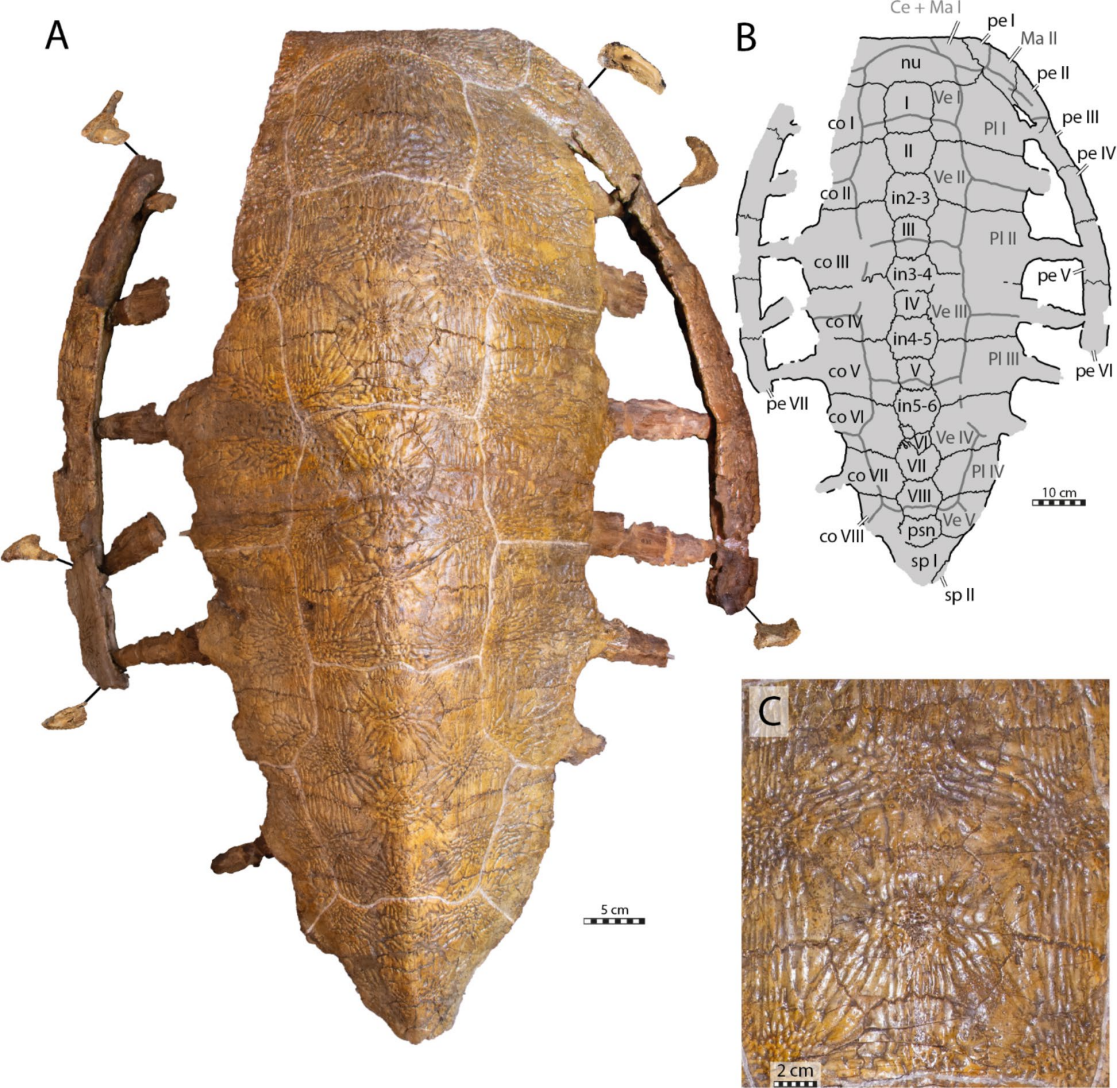
Carapace of the holotype of *Glyptochelone suyckerbuykii* (IRSNB R2), Late Cretaceous (Maastrichtian) of Netherlands. **A** Photograph in dorsal view. **B** Interpretative line drawing in dorsal view. **C**. Close up of the vertebral II area showing details of the carapacial shell texture pattern. *Ce* cervical scale, *co* costal, *in* interneural, *Ma* marginal scale, *nu* nuchal, *per* peripheral, *Pl* pleural scale, *psn* postneural, *sp* suprapygal, *Ve* vertebral scale. Roman neurals denote neurals and costal numeration

**Fig. 3:**
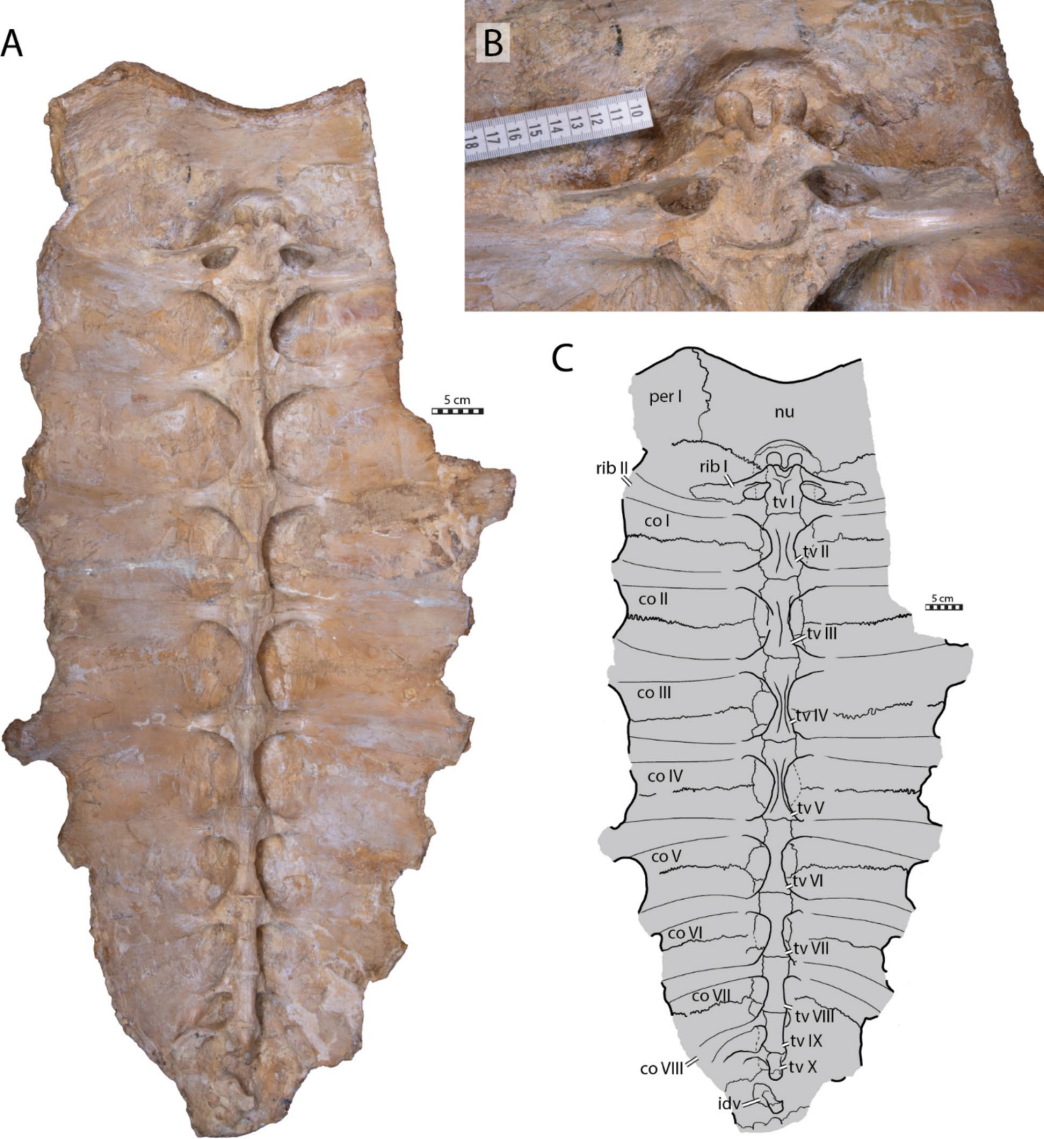
Carapace of the holotype of *Glyptochelone suyckerbuykii* (IRSNB R2), Late Cretaceous (Maastrichtian) of Netherlands. **A** Photograph in visceral view. **B** Close up showing the first thoracic vertebra and the two first ribs. **C** Interpretative drawing of the visceral view. *co* costal, *idv* indeterminate vertebra, *nu* nuchal, *per* peripheral, *tv* thoracic vertebra

**Fig. 4:**
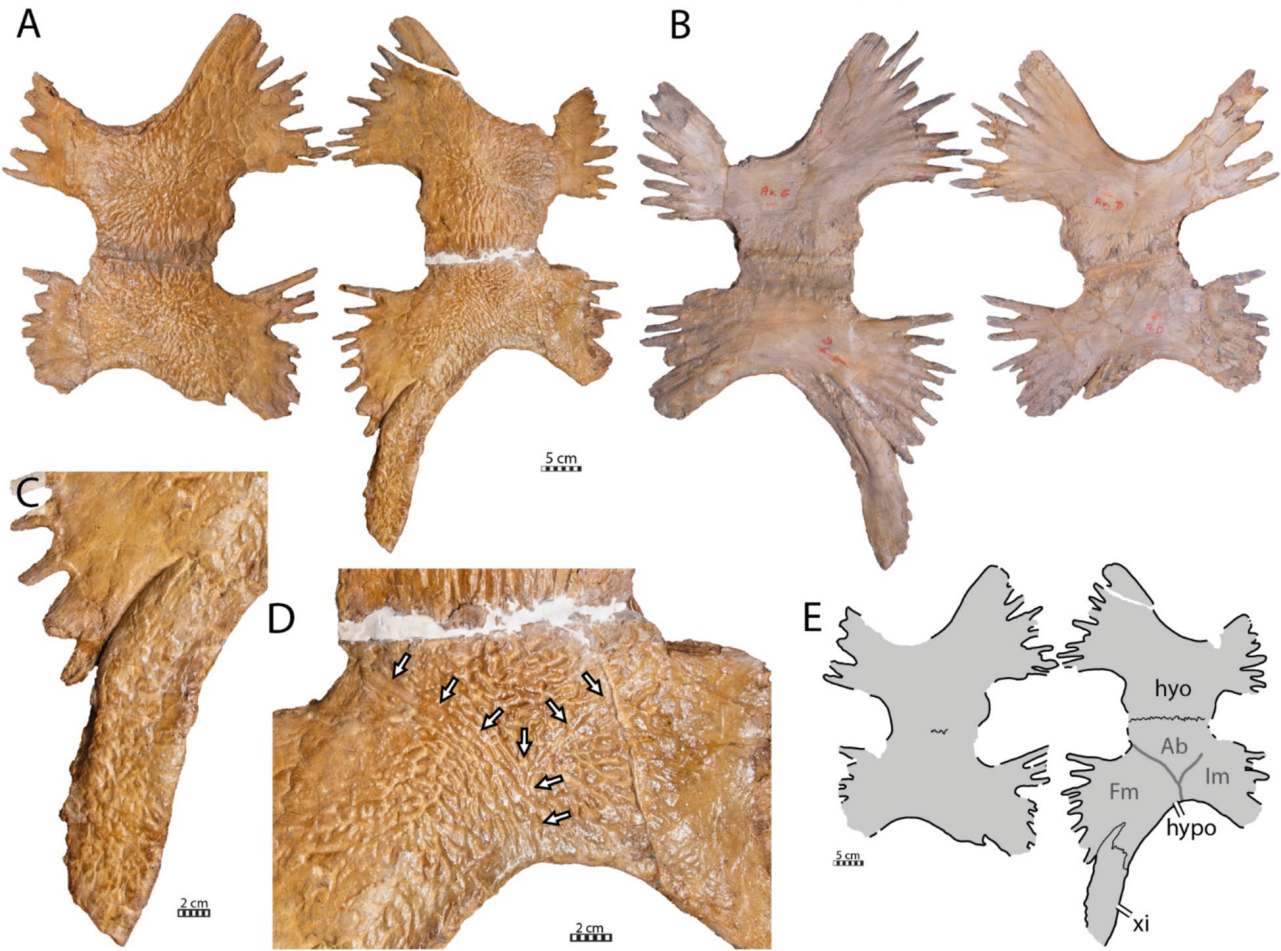
Plastron of the holotype of *Glyptochelone suyckerbuykii* (IRSNB R2), Late Cretaceous (Maastrichtian) of Netherlands. **A** Photograph of ventral view. **B** Photograph of visceral view. **C** Close up of ventral view of the xiphiplastron. **D** Close up of ventral view of the hyoplastron showing scales sulci. **E** Interpretative drawing of the ventral view. *Ab* abdominal scale, *Fm* femoral scale, *hyo* hyoplastron, *hypo* hypoplastron, *Im* inframarginal scale, *xi* xiphiplastron

**Fig. 5:**
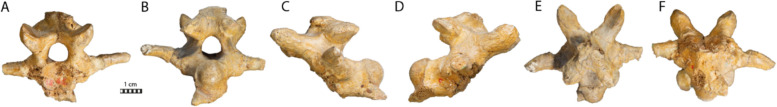
Caudal vertebra of the holotype of *Glyptochelone suyckerbuykii* (IRSNB R2), Late Cretaceous (Maastrichtian) of Netherlands. **A** anterior view. **B** posterior view. **C** left lateral view. **D** right lateral view. **E** dorsal view. **F** ventral view

**Type material.** IRSNB R2 (holotype), an incomplete shell and an isolated caudal vertebra (Ubaghs, 1879, pl. 6.1, pl.7; Minis-Van de Geijn, 1955, unnumbered text figure; Nolis et al., 2018, Fig. 4; Figs. 2, 3, 4, 5).

**Type locality.** Vilt (Ubaghs, 1879), Valkenburg aan de Geul, Limburg, the Netherlands (Fig. 1).

**Type horizon.** A few meters below the Berg en Terblijt Horizon, Meerssen Member, Maastrichtian, Late Cretaceous (Mulder & Jagt, 2016).

**Fig. 6:**
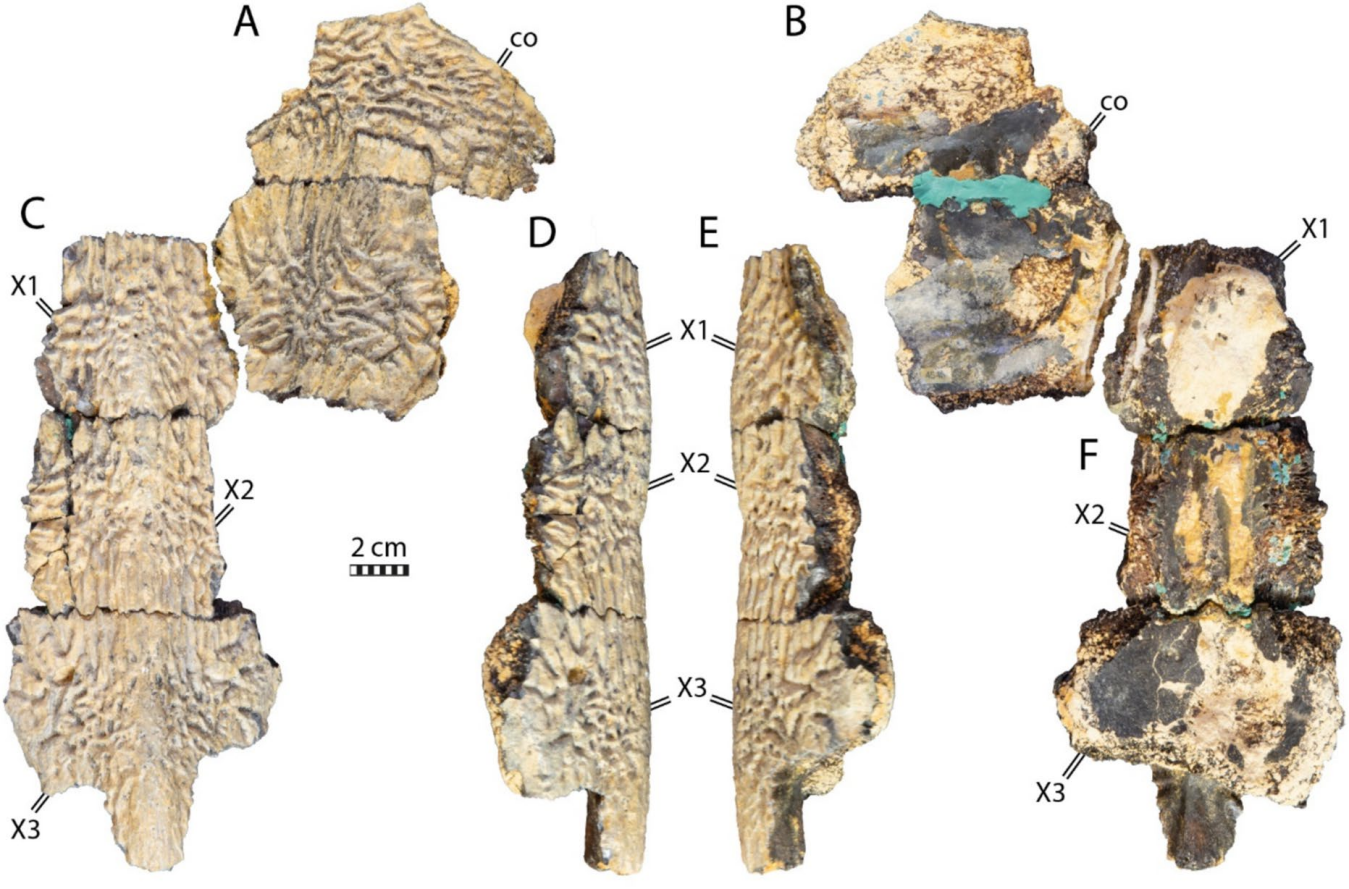
Neural and costal remains of *Glyptochelone suyckerbuykii* (NHMM 4548), Late Cretaceous (Maastrichtian) of Netherlands. Two adjoining costals in **A** dorsal and **B** ventral views. Three adjoining neurals in **C** dorsal, **D** left lateral, **E** right lateral, and **F** ventral views. *co* costal, *X1–X3* neurals elements X1–X3

**Fig. 7:**
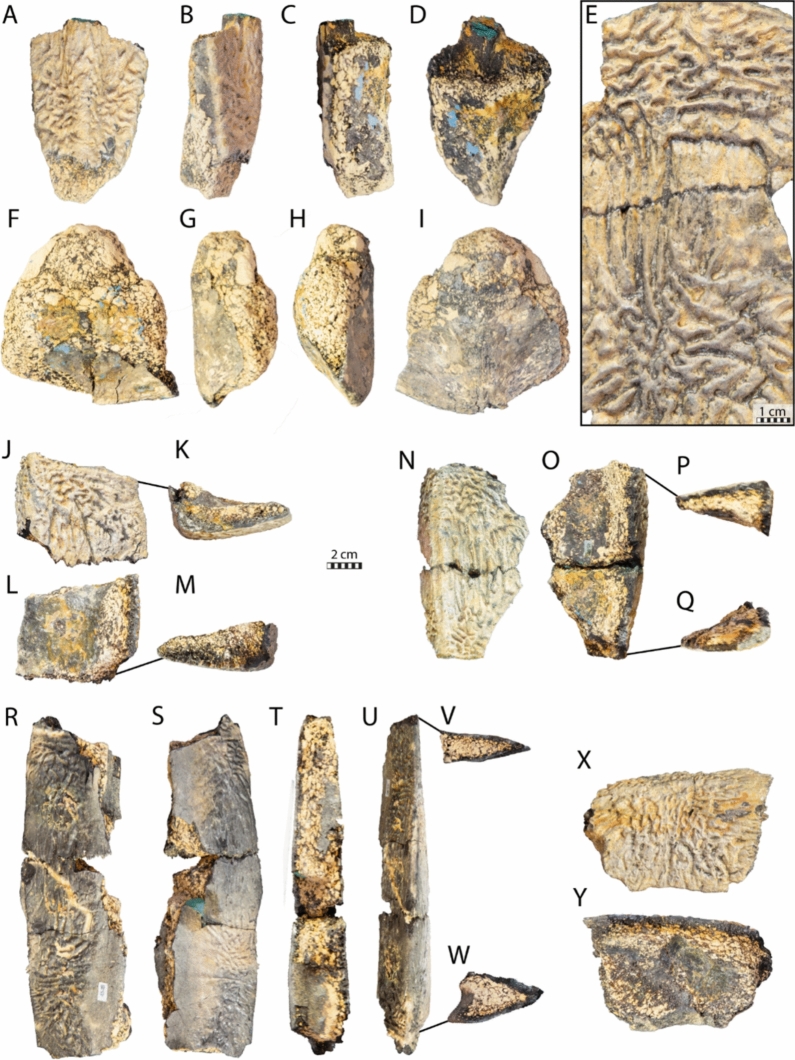
Carapace remains of *Glyptochelone suyckerbuykii* (NHMM 4548), Late Cretaceous (Maastrichtian) of Netherlands. Suprapygal 2 in **A** dorsal, **B** left lateral, **C** right lateral, and **D** ventral views. **E** Close-up view of the costals showing the texturing pattern. Pygal in **F** dorsal, **G** left lateral, **H** right lateral, and **I** ventral views. Peripheral fragment in **J** dorsal view, **K** cross section, **L** ventral view, and **M** other cross section. Peripheral fragment in **N** dorsal view, **O** ventral view, and **P** and **Q** cross sections. Two adjoining peripherals in **R** dorsal, **S** ventral, **T** medial, **U** lateral views, **V** and **W** in cross sections. Peripheral fragment in **X** dorsal, and **Y** ventral views

**Fig. 8:**
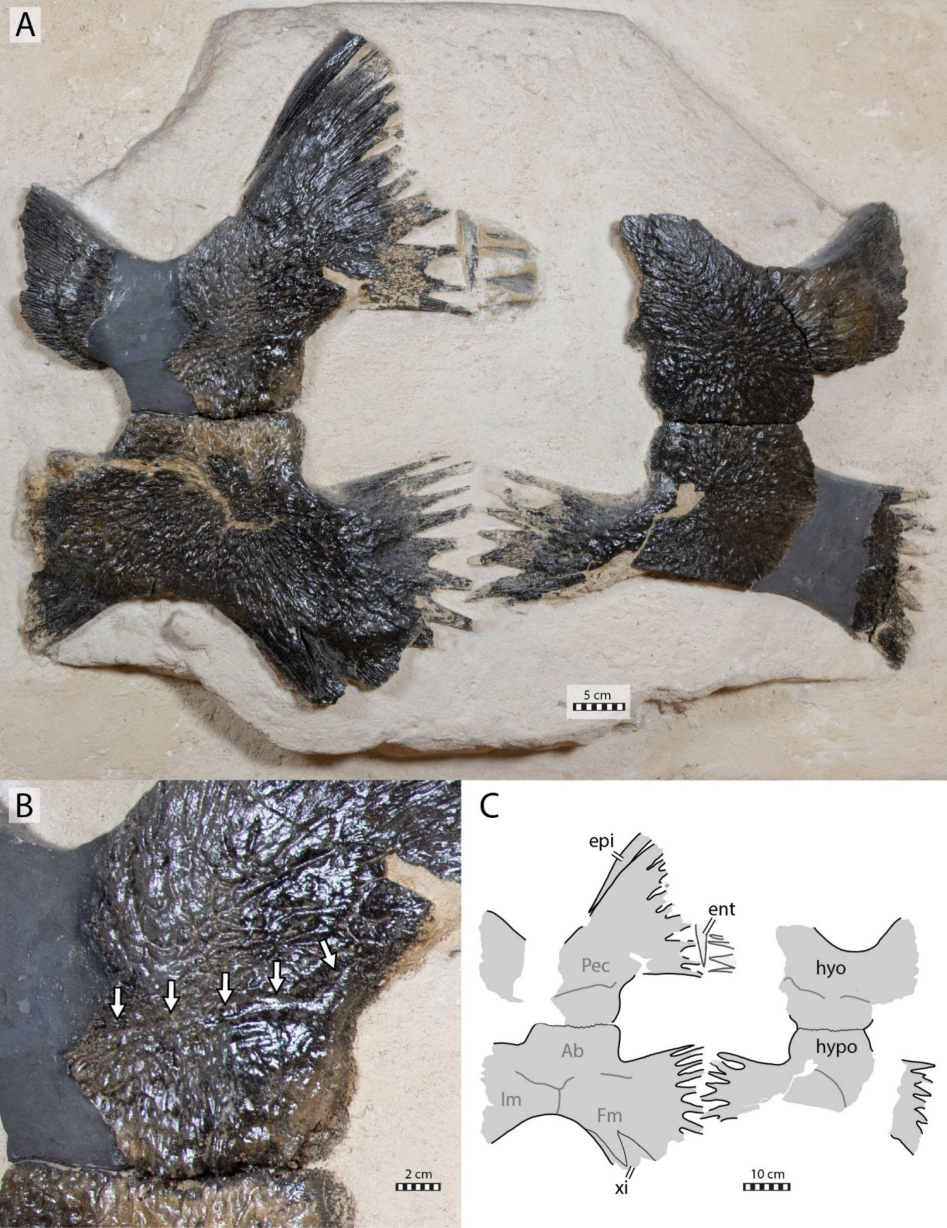
Plastron of *Glyptochelone suyckerbuykii* (NHMM 4548), Late Cretaceous (Maastrichtian) of the Netherlands. **A** Ventral view. **B** Close-up on the abdomino-pectoral scale sulcus. **C** Interpretative line drawing in ventral view. *Ab* abdominal scale, *ent* entoplastron, *epi* epiplastron, *Fm* femoral scale, *hyo* hyoplastron, *hypo* hypoplastron, *Im* inframarginal scale, *Pec* pectoral scale. Note that sutures are in thin black lines and scales in wide dark gray lines, note that the areas only preserved by imprints are in lighter gray

**Fig. 9:**
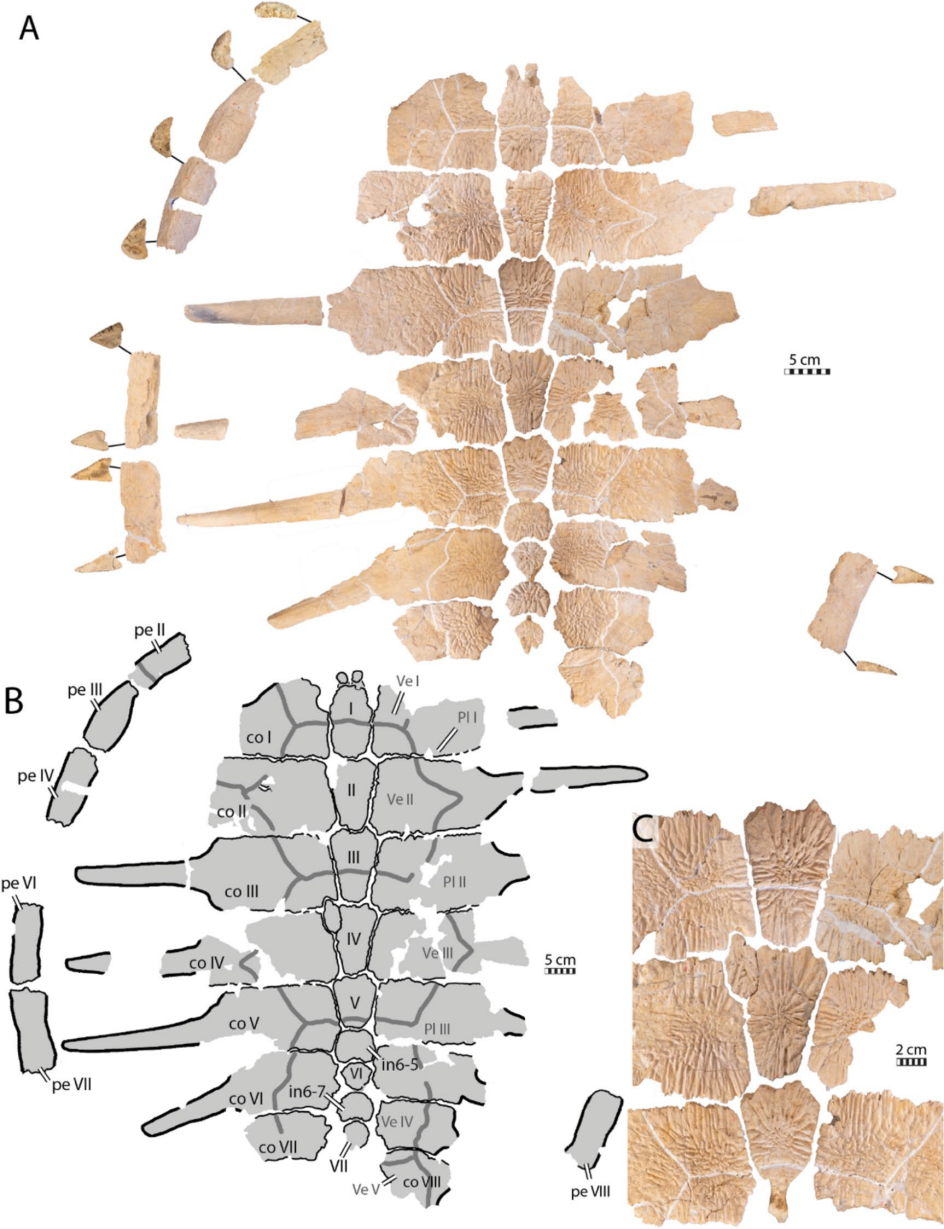
Carapace of *Glyptochelone suyckerbuykii* (IRSNB R507), Late Cretaceous (Maastrichtian) of Belgium. **A** Photograph in dorsal view with anterior and posterior views of the peripherals. **B** Interpretative line drawing in dorsal view. **C** Close up of the area of vertebral II showing ornamentation pattern. *co* costal, *per* peripheral, *Pl* pleurals scale, *Ve* vertebral scale. Roman neurals denote neural elements

**Fig. 10:**
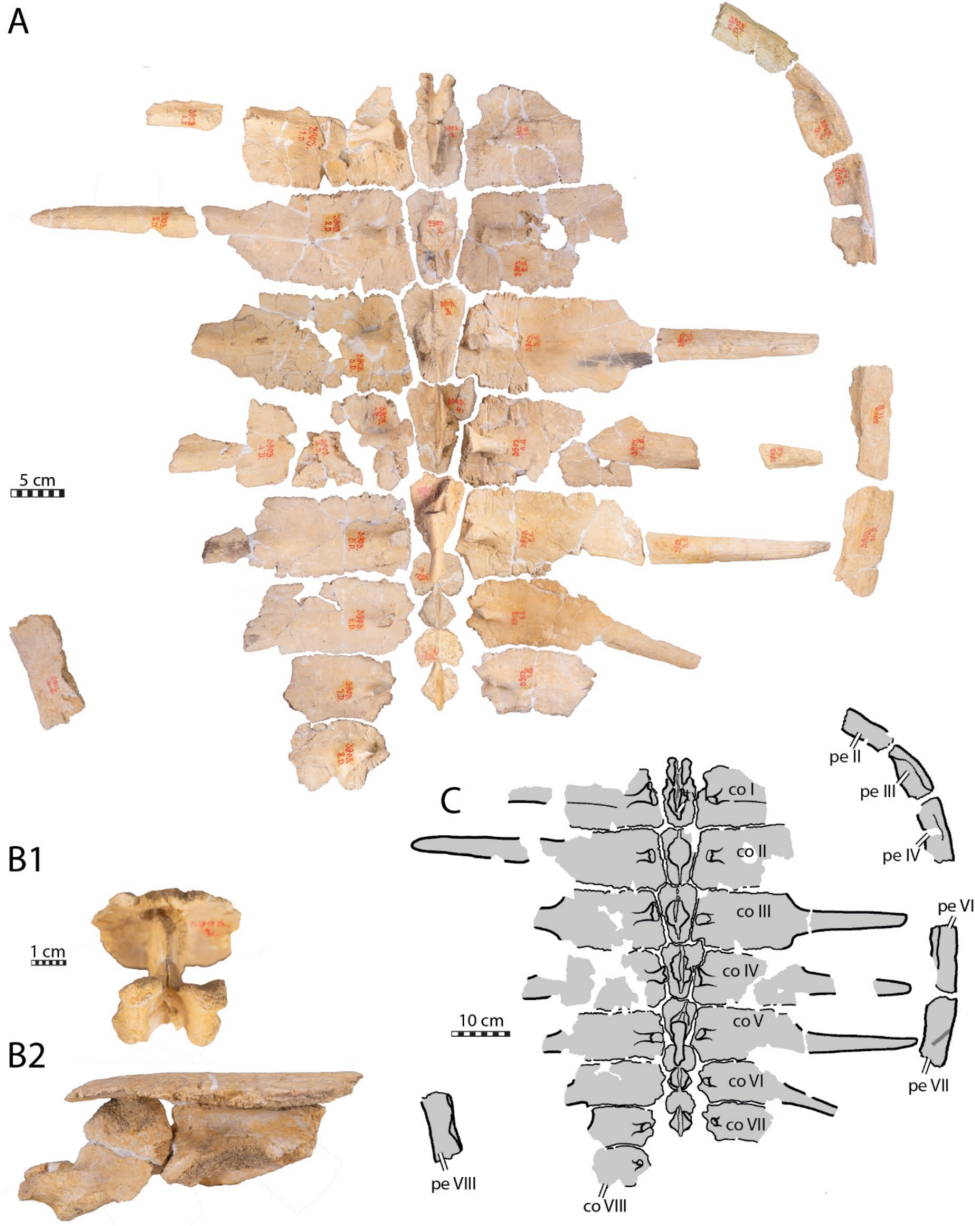
Carapace of *Glyptochelone suyckerbuykii* (IRSNB R507), Late Cretaceous (Maastrichtian) of Belgium. **A** Photograph of visceral view. Photographs of the first thoracic vertebra in **B1** anterior and **B2** lateral left view. **C** Interpretative line drawing of the visceral view. *co* costal, *pe* peripheral

**Fig. 11:**
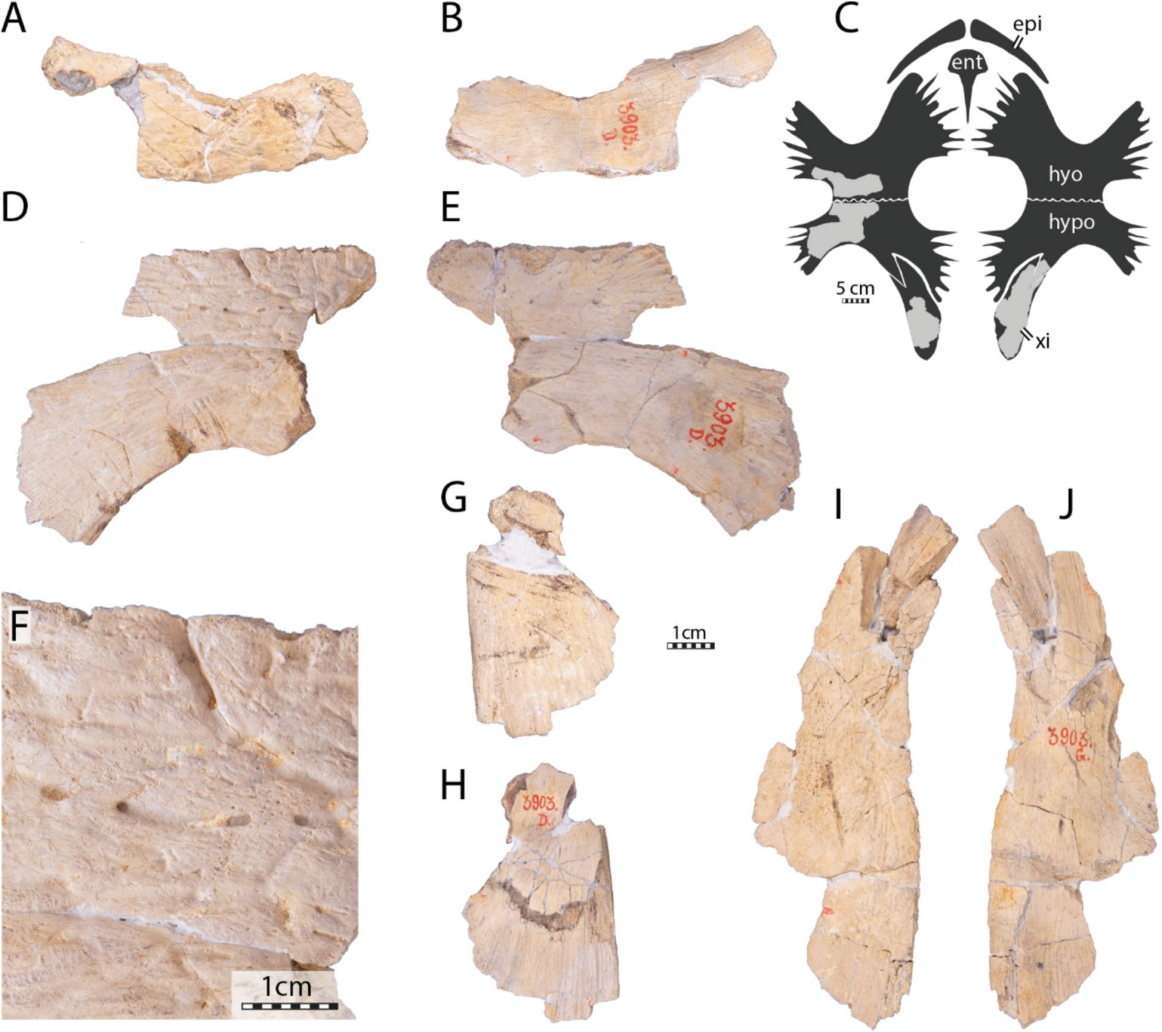
Plastron of *Glyptochelone suyckerbuykii* (IRSNB R507), Late Cretaceous (late Campanian) of Belgium. **A** Photograph of right hyoplastron in ventral view. **B** Photograph of right hyoplastron in visceral view. **C** Interpretative drawing showing preserved parts in ventral view. **D** Photograph of right hypoplastron in ventral view. **E** photograph of right hypoplastron in visceral view. **F** Close up of hyoplastron, highlighting ornamentation. **G** Photograph of right xiphiplastron in ventral view. **H** Photograph of right xiphiplastron in visceral view. **I** Photograph of left xiphiplastron in ventral view. **J** Photograph of left xiphiplastron in visceral view. *ent* entoplastron, *epi* epiplastron, *hyo* hyoplastron, *hypo* hypoplastron, *xi* xiphiplastron

**Fig. 12:**
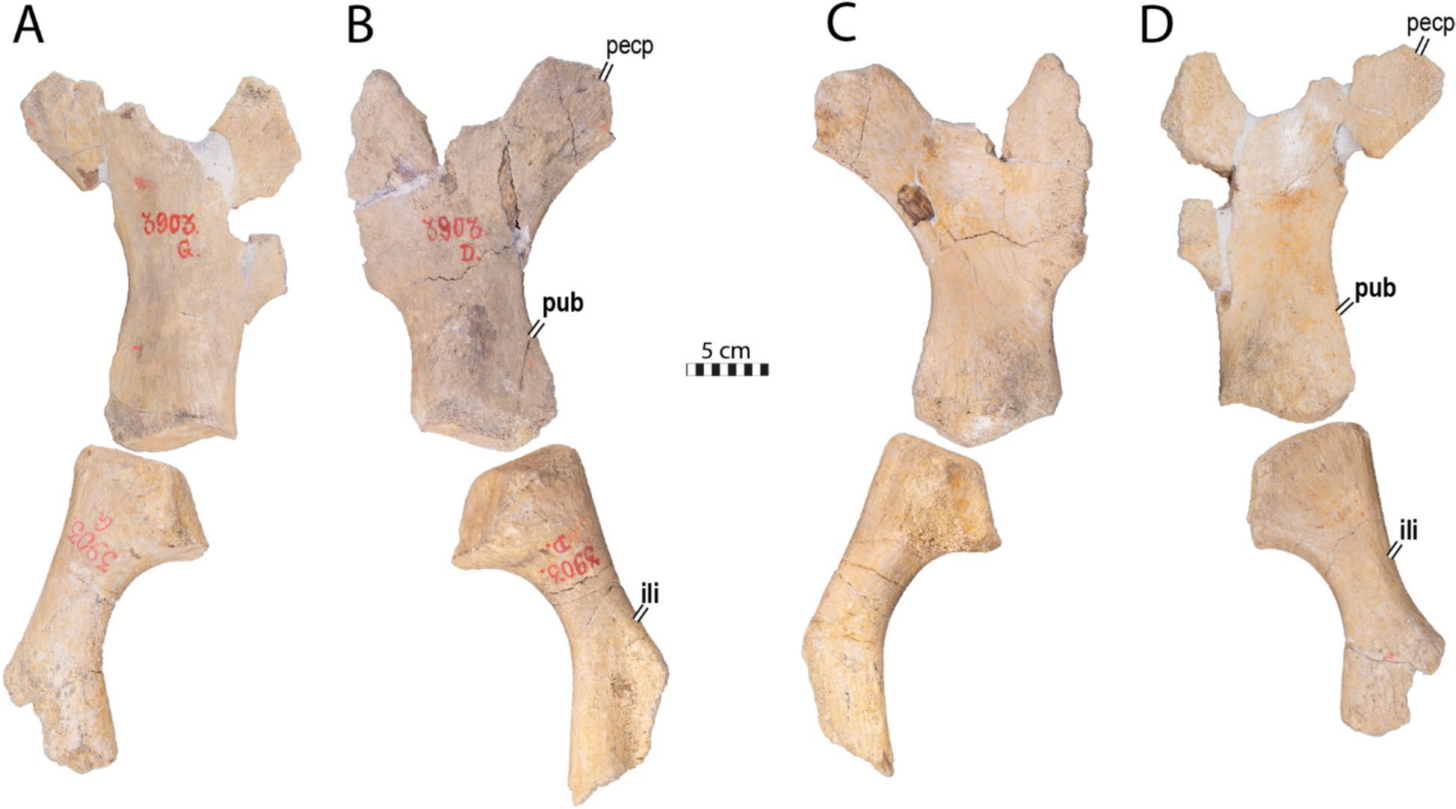
Photograph of pelvic remains of *Glyptochelone suyckerbuykii* (IRSNB R507), Late Cretaceous (Maastrichtian) of Belgium. **A** Left pubis and ilium in dorsal view. **B** Right pubis and ilium in dorsal view. **C** Right pubis and ilium in ventral view. **D** Left pubis and ilium in ventral view. *ili* ilium, *pecp* pectineal process, *pub* pubis. Note that bones are labelled in bold

**Fig. 13:**
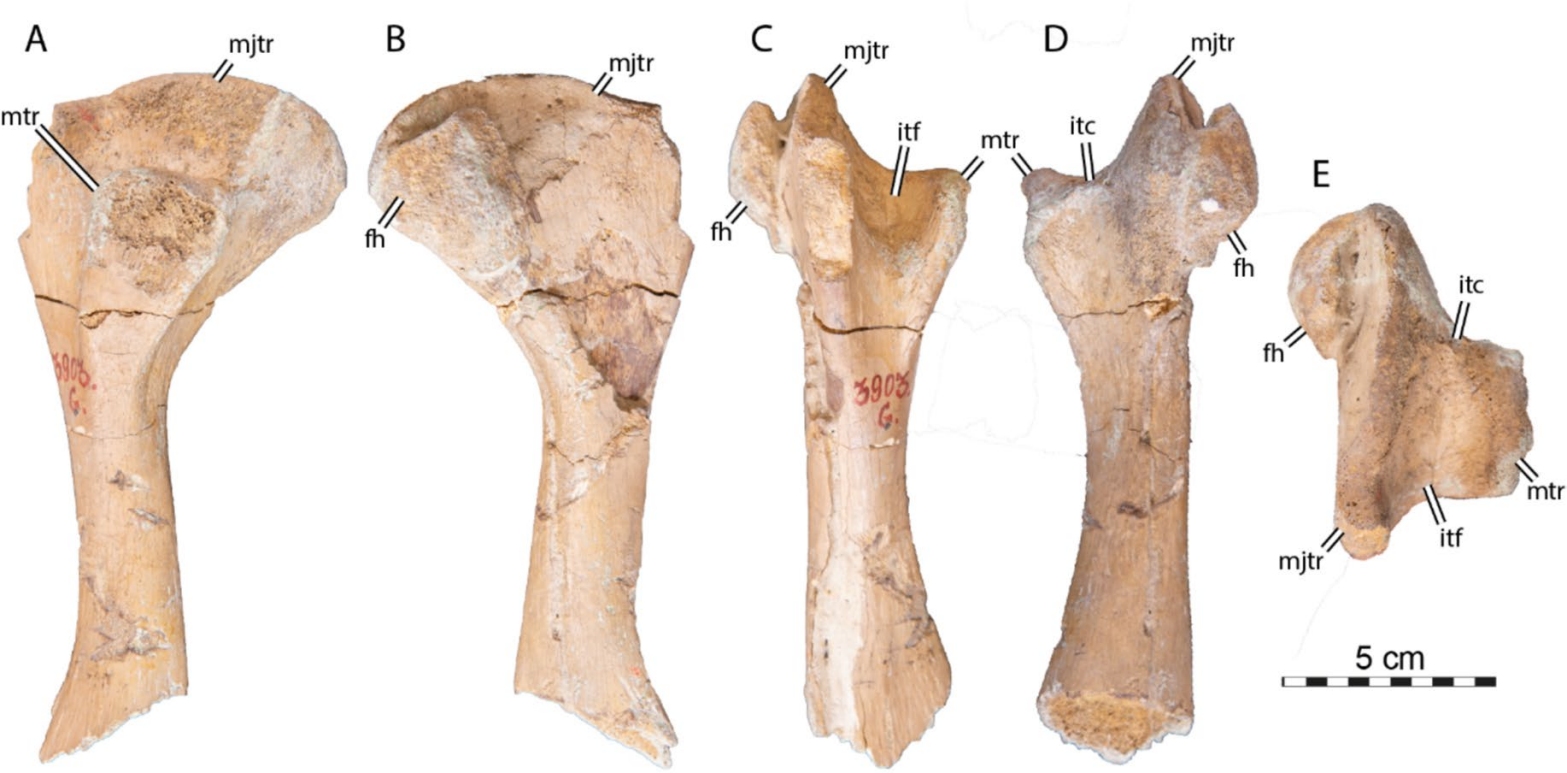
Photograph of the proximal left femur in standardized views of *Glyptochelone suyckerbuykii* (IRSNB R507), Late Cretaceous (late Campanian) of Belgium. **A** Anterior view. **B** Posterior view. **C** Ventral view. **D** Dorsal view. **E** Proximal view. *fh* femoral head, *itc* intertrochanteric crest, *itf* intertrochanteric fossa, *mjtr* major trochanter, *mtr* minor trochanter

**Referred material.** NHMM 4548, isolated neurals, costal fragments, several isolated peripherals, a suprapygal II, a pygal, and a fairly complete plastron (Kruytzer, 1955, Fig. 2; Figs. 6, 7, 8) from the former quarry Van der Zwaan, Kalkmergel Mij, Sint-Pietersberg, the Netherlands, upper Meerssen Member, latest Maastrichtian, Late Cretaceous (Mulder et al., 1998; Fig. 1); IRSNB R3, an isolated neural (Ubaghs, 1879, pl. 6.2, unfigured herein) from Kanne, Belgium, Maastricht Formation, latest Maastrichtian, Late Cretaceous (Felder, 1980; Mulder et al., 1998; Fig. 1); IRSNB R507, an incomplete shell, proximal left femur, both pubes and ilia (Figs. 9, 10, 11, 12, 13) from Spiennes, Belgium, Spiennes Chalk, latest Campanian, Late Cretaceous (Keutgen et al., 2011; Fig. 1).

**Diagnosis.** *Glyptochelone suyckerbuykii* can be distinguished from other Late Cretaceous pan-chelonioids by its distinct shell surface texture consisting of radiating crests and vermiculations associated with large spheric foramina, presence of interneurals, and the complete absence of a nuchal pedestal.

## Description and comparison

### IRSNB R2

The holotype of *Glyptochelone suyckerbuykii* (IRSNB R2) consists of a nearly complete shell, including most of the nuchal, the full neural series with associated thoracic vertebrae, eight pairs of costals with minor damage to left costals I and II, left peripherals III–VII, right peripherals I–VI, a nearly complete first suprapygal (Figs. 2, 3), the right and left hyo and hypoplastra, the left xiphiplastron (Fig. 4), and an isolated caudal vertebra (Fig. 5).

The overall shape of the shell is elongated (Fig. 2A, B) with a length of about 96 cm from the nuchal to suprapygal I and a width of about 70 cm at the level of the contacts of peripherals V and IV. The shell displays extensive costoperipheral and plastral fontanelles. Among Late Cretaceous pan-chelonioids, this condition reminds of *Ctenochelys stenopora* (Zangerl, 1953a) and *Allopleuron hofmanni* (Mulder, 2003), and *Euclastes*-like chelonioids (Ullmann & Carr, 2021; Zangerl, 1953a) but differs from *Porthochelys laticeps* and some specimens of *Toxochelys latiremis* (Zangerl, 1953a), which lack extensive fontanelles.

The thickness of the bones varies greatly across the shell, from around 1.5 cm at the anterior nuchal border, which is rather thick for a sea turtle, to around 4 mm at the intercostal sutures. The shell of IRSNB R2 is not keeled and lacks epineural ossifications. The carapace displays well marked scale sulci on its dorsal face (Fig. 2). Its entire external surface, with exception of the free rib ends, is covered by a deep ornamentation formed by fine vermiculations and straight crests that notably radiates from the ossification center of each bony plate (Fig. 2A, C). At the center of each bone, numerous pore-like foramina are apparent, of which the largest are approximate 2 mm in diameter (Fig. 2C).

The plastron of IRSNB R2 displays the same vermiculated radiating pattern but the central foramina are less numerous than on the carapace (Fig. 4). Despite the excellent preservation of the ventral face of the plastron, we can only discern the paths of the left abdomino-femoral and the most posterior abdomino-inframarginal sulci (Fig. 4D, E).

#### Nuchal

The nuchal of IRSNB R2 is nearly complete, but its left lateral aspects are missing (Figs. 2A, B, 3). The state of preservation is nonetheless sufficient to appreciate its overall shape. The nuchal is a thick and wide, pentagonal bony plate that forms the median part of the anterior edge of the carapace. The nuchal contacts the first peripheral anterolaterally, the first costal posterolaterally, and neural I posteromedially. It resembles *Asmodochelys parhami* (Gentry et al., 2019) by being wide relative to the width of the shell and differs strongly from the Santonian stem-chelonioids *Toxochelys latiremis* and *Porthochelys laticeps*, which display a much narrower nuchal relative to shell width (Zangerl, 1953a).

In IRSNB R2, the nuchal emargination is present but shallow. This condition differs from that of *Allopleuron hofmanni* and *Asmodochelys parhami*, which have deep nuchal emarginations (Gentry et al., 2019; Mulder, 2003), but resembles that of *Peritresius ornatus* (Baird, 1964).

Postnuchal fontanelles are notably absent in IRSNB R2. This contrasts many Late Cretaceous pan-chelonioids, including *Ctenochelys stenopora*, *Peritresius ornatus*, and *Toxochelys latiremis* (Baird, 1964; Zangerl, 1953a).

A notable feature of IRSNB R2 is the thickened and rounded anterior margin of the nuchal, which is about 2 cm thick, far greater than the rest of the nuchal. This condition resembles that seen in *Allopleuron hofmanni* (Mulder, 2003) and *Peritresius ornatus* (Baird, 1964) but is absent in many Cretaceous pan-chelonioids, such as *Toxochelys latiremis* (Gentry & Ebersole, 2018; Nicholls, 1988; Zangerl, 1953a), *Ctenochelys stenopora* (Matzke, 2007; Zangerl, 1953a), and *Euclastes*-like turtles (Parris et al., 1986; Schmidt, 1944).

The ornamental surface pattern is not uniform on the dorsal surface of the nuchal of IRSNB R2 (Fig. 2A). Indeed, towards the midline of the anterior nuchal margin, the crests become shorter, more vermiculated and tend to disappear, in contrast to the posterolateral edges of the bone, which display long, deep, and nearly straight crests.

The visceral face of the nuchal of IRSNB R2 displays a deep and rounded pocket at its posterior median margin, which likely accommodated the eighth cervical vertebra (Fig. 3). This pocket extends onto neural I and the medial margin of costal I and is about as wide as the costovertebral tunnel, with which it is confluent. In *Allopleuron hofmanni* (see NHMM 9016), a wide depression is present in this area, but it is less marked and affects only the posterolateral margin of the visceral face of the nuchal.

A pedestal for the last cervical is notably absent in IRSNB R2 (Fig. 2A, C), contrasting with *Asmodochelys parhami* (Gentry et al., 2019), *Euclastes wielandi* (see NJSM 12295), *Prionochelys matutina* (Gentry, 2018), *Toxochelys latiremis* (Nicholls et al., 1990), and modern chelonioids. In *Allopleuron hofmanni*, an articulation site for the eighth cervical is present on the visceral surface of the nuchal, but it is developed as an anteroposteriorly elongated pit bordered by low, slightly elevated bulges (Mulder, 2003; see also Additional file 8, Fig. 1D). This condition, too, is absent in IRSNB R2, which displays a perfectly flat surface on the ventral side of the nuchal.

#### Preneural

A preneural is absent in IRSNB R2 (Fig. 2A, C), as in most fossil pan-chelonioids. This feature is present in some specimens of *Ctenochelys stenopora* (Matzke, 2007; Zangerl, 1953a) and occurs in some extant cheloniids as intraspecific variation (Pritchard, 1988; Fig. 16D, E).

#### Neural series

The neural series of IRSNB R R2 includes 13 neural elements (Fig. 2A, B), which is unusual for turtles (Pritchard, 1988). These 13 bony plates do not all correspond to genuine neurals because some lack a contact with the neural arches (Figs. 3C, 16A–C), as previously noticed by Ubaghs (1879) and Zangerl (1960). We here designate these elements as interneurals (see Discussion). The presence of interneurals in the neural series of IRSNB R2 was previously interpreted as a consequence of the secondary subdivision of true neural plates during ontogeny (Kordikova, 2002; Pritchard, 1988; Zangerl, 1960, 1969, 1988; Zangerl & Turnbull, 1955). Although it is possible that this condition is the result of a pathology, the neural series of IRSNB R2 appears to be quite symmetric, except for the sixth neural, the tenth element of the neural series, which is irregularly shaped and associated with a small, pathological supernumerary element on its left side. The neural series of IRSNB R2 notably contrasts that of IRSNB R507, which only includes ten neurals elements, of which the first five are regular neurals notably longer than wide (see description below). Neural series including more than ten elements due to the presence of interneurals are otherwise known from some Cenozoic chelonioid sea turtles, such as *Procolpochelys grandaeva* (Zangerl & Turnbull, 1955), *Procolpochelys charlestonensis* (Weems & Brown, 2017), and *Euclastes melii* (Zangerl, 1960), but this also occurs in some individuals of modern cheloniids, in particular in *Lepidochelys kempii* and *Lepidochelys olivacea* (Deraniyagala, 1939; Kordikova, 2002; Pritchard, 1988; Zangerl, 1969; Zangerl & Turnbull, 1955; Additional file 8, Fig. 2), with some individuals displaying a fairly symmetrical neural series that consists of up to fifteen neural elements (e.g., Deraniyagala, 1939, Fig. 60, see Discussion). No other Cretaceous pan-chelonioid, indeed turtle, is known to display this morphology.

All neural elements of IRSNB R2 are as wide as long (Fig. 2). A similar arrangement is seen in other species with interneurals like *Procolpochelys grandaeva* (Weems, 1974; Zangerl & Turnbull, 1955) and *Procolpochelys charlestonensis* (Weems & Brown, 2017). Wide neurals also occur in some Cretaceous taxa without interneurals, such as *Asmodochelys parhami* (Gentry et al., 2019), *Peritresius ornatus* (Baird, 1964), and *Allopleuron hofmanni* (Mulder, 2003), but in these taxa this widening is a result from the broadening of the neural series.

The epineurals found in ctenochelyids (Baird, 1964; Gentry, 2018; Gentry et al., 2019; Zangerl, 1953a) are absent in IRSNB R2.

In contrast to *Allopleuron hofmanni* (Mulder, 2003) and most Late Cretaceous protostegids, including *Archelon ischyros* (Wieland, 1904; Zangerl, 1953b), *Calcarichelys gemma* (Hooks, 1998; Zangerl, 1953b), *Mesodermochelys undulatus* (Hirayama & Chitoku, 1996; Nakajima et al., 2011), and *Protostega gigas* (Zangerl, 1953b), a crisp median keel is absent. Instead, the shell of IRSNB R2 displays a broad medial ridge from neural VI to suprapygal I that gives the posterior aspects of the shell a rounded, tectiform shape (Fig. 2A).

The neural formula of IRSNB R2, including the postneural, is 4, 6 A, I6P, 4, I6, 4, I6, 4, I6A, 4, 6 A, 6, 4, whereby the I’s designate interneurals (see Discussion for proposed nomenclature, Figs. 2B, 11B, 16C, 17). Neurals I, III, IV, V, and the postneural are roughly quadrangular and entirely surrounded laterally by costals I, III, IV, V, and VIII respectively (see Discussion, Figs. 16A–C, 17A). The neurals that are laterally surrounded by one pair of costals are significantly smaller than the remaining neural elements, as in *Procolpochelys charlestonensis* (Weems & Brown, 2017). In most Late Cretaceous pan-chelonioids, only a single, square neural is present, either the ninth/postneural (e.g., *Lytoloma angusta* Wieland, 1904; *Euclastes wielandi* NJSM 12295; *Ctenochelys stenopora* Zangerl, 1953a; also see YPM 1786) or the first neural (*Allopleuron hofmanni* Mulder, 2003; *Prionochelys matutina* Gentry, 2018; *Asmodochelys parhami* Gentry et al., 2019). As in *Toxochelys moorevillensis* (FMNH PR28) or *Peritresius ornatus* (Baird, 1964), the remaining neural elements of IRSNB R2 are hexagonal (Fig. 2A, B), in particular neurals II, VII, and VIII and interneurals 2/3, 3/4, 4/5, and 5/6 (Figs. 2C, 11, 17D). An aberrant, triangular element is wedged between interneural 5/6 and neural VI giving both elements an irregular outline.

The visceral face of the shell of IRSNB R2 preserves the complete thoracic vertebral series in articulation with the neural series (Figs. 3A, C, 16A, B). The thoracic series, as in most turtles, contains ten elements consisting of a first, short, and procoelous vertebra, followed by eight platycoelous vertebrae, and, finally, a last, tenth, procoelous thoracic vertebra. The first thoracic vertebra is shorter than the others but remains longer than wide. The first thoracic prezygapophyses have anteriorly descending dorsal surface that are oriented obliquely (Fig. 3B). Unfortunately, the central articulation with the eight cervical is reconstructed, not preserved, making it impossible to observe its orientation. The other thoracic vertebrae of IRSNB R2 display elongated centra, which become strongly constricted at mid length. This constriction is particularly well developed for vertebrae III to VI.

The centra of thoracic vertebrae II to V exhibit a well-marked, ventral keel (Fig. 3A, C). This condition differs from that of *Allopleuron hofmanni* (see NHMM 9016), in which thoracic centrum III is devoid of a keel and centra II, IV, and V display only a low keel that is difficult to discern. As in most turtles, the first thoracic vertebra of IRSNB R2 does not form a neural plate (Kordikova, 2000). Thoracic vertebrae II–IX display dorsal expansions that correspond to neurals I–VIII (i.e., the first, second, fourth, sixth, seventh, tenth, eleventh and twelfth neural elements of the series). The possible presence of a sutural contact between the neural arch of the tenth thoracic vertebra and the thirteenth midline element cannot be evaluated with certainty due to the presence of matrix in this area, but the presence of a median bone expansion situated at the visceral side of the thirteenth element, anterior to the tenth rib, is suggestive of a probable sutured contact. This differentiates the thirteenth element from a suprapygal, which lack a contact with thoracic neural arches altogether (Additional file 8, Fig. 4), as well as from a true neural, which is an outgrowth of the thoracic neural arch itself and thus lacks a suture, which are indicative of separate ossifications. It is for this reason that we identify this neural element as a postneural [i.e., an element situated posterior to the neural series that does not represent a true neural (i.e., an outgrowth of a thoracic neural) but displays a secondary contact with the tenth thoracic neural arch via a sutural connection; see Discussion for nomenclature]. A similar contact occurs sometimes in modern cheloniids, in particular in *Chelonia mydas* (Pritchard, 1988), in which the element posterior to the eighth neural has a tendency to expand throughout ontogeny to reach the neural arch. This secondary contact can already occur at a juvenile stage (Fig. 16D, E).

Thoracic ribs II and III are placed at the level of the intervertebral contacts. Their median articulations with the vertebrae are therefore split evenly. The more posterior thoracic ribs increasingly shift posteriorly, thus increasing their medial contact with the more posterior thoracic vertebra. Yet, unlike many recent turtles, no thoracic rib exhibits a single medial contact with a thoracic vertebra. Modern cheloniids display a morphology that reminds of that found in IRSNB R2. The spaces between the thoracic centra II, VII and IX and the visceral face of the neural series are filled along the midline by a mixture of laminar bone and matrix. These “laminae” are potentially formed by the anterior and posterior ends of the dorsal spines of the neural arches, which have a tendency in modern cheloniids to expand anteroposteriorly to contact one another above the thoracic centra (see Additional file 8, Fig. 2B).

#### Costals

The shell of IRSNB R2 preserves eight pairs of costal plates, but the free distal rib ends are partially damaged (Fig. 2A, B). Furthermore, left costals I and II are broken, with only their medial halves remaining preserved. The costoperipheral fontanelles are well developed but their width is less than that of the ossified costal plate. The flattened free rib ends are about one third of the anteroposterior length of the costal plates. The lateral margins of the costals taper before grading into the free end, reminiscent of the condition found in *Prionochelys nauta* (Zangerl, 1953a). Consequently, the costoperipheral fontanelles of IRSNB R2 form rounded rectangles, a condition that is intermediate between what is found in some protostegids, where the fontanelles taper more progressively to form triangular fontanelles (e.g., *Chelosphargis advena* and *Protostega gigas* [Zangerl, 1953b]), and the condition of *Allopleuron hofmanni*, in which the ribs emerge suddenly from the costals, resulting in rectangular fontanelles (Mulder, 2003). In addition, IRSNB R2 lacks the elongated, rod-like processes of the costal callosity that covers the rib ends of *Allopleuron hofmanni* (see Additional file 8, Fig. 1). The costal plates are quite thin, around 4 mm thick at their anterior and posterior borders. The costals gradually become anteroposteriorly shorter towards the posterior, with costal VIII being approximately half the length of costal I (Figs. 2A, B, 3A, C). Most thoracic rib heads are preserved (Fig. 3A, C). As is common in fossil pan-chelonioids, the first thoracic rib of IRSNB R2 is reduced in size, with a diameter and length approximately one-third that of the second thoracic rib (Zangerl, 1953a). The first thoracic rib merges with the second thoracic rib slightly distal to the latter’s coalescence with the first costal callosity (Fig. 3A, B). The first thoracic rib head is triangular and contacts the anterior part of the first thoracic vertebra. Rib heads II to VI are rather wide and share an extensive contact with the adjacent thoracic vertebrae, whereas rib heads VII to IX become increasingly smaller towards the posterior (Fig. 3A, C). In visceral view, costal plates II to VII are smooth lateral to the emergence of the rib head, making it difficult to track the rib on the visceral face on these bones. The remaining ribs are more easily distinguished from the costal callosities. While thoracic ribs II–VIII are mostly oriented laterally, thoracic rib IX is strongly curved to the posterior, suggesting presence of a rib free peripheral X. Thoracic rib X is small, like rib IX, but can be traced more distally on the visceral face of the costal VIII and displays a curved shape that is concave anteriorly.

#### Suprapygal region

The suprapygal region of IRSNB R2 is affected by several cracks, complicating interpretation of its morphology (Figs. 2A, B, 3A, C). Additionally, the sutures are not clearly visible on the visceral face of this area. The thirteenth element of the neural series (Fig. 2A, C) contacts the posterior half of the medial rim of costal VIII and seems to contact the neural arch of the tenth thoracic vertebrae even if the potential suture is obscured by matrix (see above). Therefore, this element is here interpreted as a postneural, rather than a suprapygal, which lack thoracic neural arch contacts altogether (see Discussion).

The first true suprapygal is pentagonal with long posterolateral sides. The ornamentations, indicative of a single ossification center, radiate from the center of the preserved suprapygal region (Fig. 2A). A fragment of suprapygal II seems to remain articulated on the right posterolateral side of suprapygal I (Fig. 2A, C). Suprapygal I is affected by the low median dorsal ridge that gives the carapace its tectiform shape.

#### Peripherals

The peripheral ring of IRSNB R2 is incompletely preserved, with only the right peripherals I to VI and left peripherals III to VIII remaining in articulation with the main body of the shell (Fig. 2A, B). Fragments we interpret as the posterior half of the left sixth and the anterior two-thirds of the left seventh peripherals are available but not figured in the main text (Additional file 8, Fig. 4), as they are too fragmentary and have undergone deformation. The dorsal face of the peripherals of IRSNB R2 (Fig. 2A, B) lacks the gutter-like-ridge seen in *Allopleuron hofmanni* (Mulder, 2003) and some ctenochelyids (e.g., *Peritresius martini*, Gentry et al., 2018). The dorsal and ventral faces of the peripherals are covered by the same texture as the rest of the shell. Peripheral I contacts the nuchal posteromedially, the first costal posteriorly, and the second peripheral laterally. This bone is roughly triangular in dorsal view and does not contribute to the shallow nuchal emargination but instead forms a low lateral bump at the lateral sides of the nuchal. In cross section, the anterior margin of the second peripheral is banana-shaped, while the posterior margin is triangular with an angle of about 70° (Fig. 2A). The resulting deep medial visceral groove runs along the medial face of peripherals III to VII but becomes deeper and narrower posteriorly, where the angle between the dorsal and ventral walls of the peripherals decreases progressively. Consequently, as is usual in sea turtles, the peripherals become flatter posterior to peripheral V (Fig. 2A). The medial face of peripherals III to VII are also marked by small, shallow, and ovoid rib insertion pits for articulation with thoracic ribs I to VI, respectively (Additional file 8). The second rib pit insertion of IRSNB R2 is situated at the limit between the second and third peripherals, while the insertion pits III to VI are situated at the middle of their corresponding peripherals.

#### Carapace scales

Scale sulci are generally well preserved on the carapace of IRSNB R2, but the marginal sulci can barely be discerned, mostly due to the damaged external surfaces of the peripherals (Fig. 2A, B).

All recent cheloniids possess at least one cervical that is restricted to the nuchal. Although we can discern a sulcus-like structure on the right side of IRSNB R2, we are not able to find a similarly placed structure on the left side of the specimen (Fig. 2A, B). This either implies that a cervical was absent entirely, or that a cervical was present, but did not form distinct lateral sulci, or that the cervical had fused with the adjacent first marginal during ontogeny. The available data is insufficient to conclusively support any particular hypothesis. The joint area covered by the cervical and first marginal is bilobate, laps onto the medial third of peripheral I, posteriorly embraces vertebral I, posterolaterally contacts pleural I, and laterally contacts marginal II.

The vertebral scale series is well preserved. Neurals I, III, V and VIII are respectively crossed by the posterior limits of the vertebral scales I, II, III and IV (Fig. 2A, B). The vertebral series of IRSNB R2 is moderately wide, with vertebrals I to III only covering the medial third of the costals plates. Vertebral I is wider than long, displays a long and anteriorly concave rounded anterior sulcus, a sinuous lateral sulcus, and a posteriorly concave sulcus. Vertebral II and III are hexagonal elements and clearly longer than wide. Vertebral IV is octagonal and much longer than wide. Its anterolateral sides form an obtuse angle of about 150°. Vertebral V was likely pentagonal.

Costals II, IV, and VI of IRSNB R2 are crossed by the straight, relatively transverse posterior sulci tracks of the pleurals I, II, and III, respectively (Fig. 2A, B). The posterior sulcus of pleural IV crosses costal VIII at an angle of around 40°.

#### Plastral bones

The hyoplastra, hypoplastra, and the left xiphiplastron are preserved, but the epiplastra and the entoplastron are missing (Fig. 4). The available bones are well preserved, including their textured surface. However, most of the scale sulci are indistinct, although a few conspicuous tracks prove the presence of plastral scales. Plastral scale sulci are present in the basal pan-chelonioid *Toxochelys latiremis* (Gentry & Ebersole, 2018; Zangerl, 1953a), the ctenochelyids *Ctenochelys stenopora* (Matzke, 2007), *Peritresius martini* (Gentry et al., 2018), and *Peritresius ornatus* (Baird, 1964), and the “euclastesid” *Catapleura arkansaw* (Schmidt, 1944), but notably absent in *Allopleuron hofmanni*.

The lateral and central fontanelles are well developed and display an overall rectangular shape, contrasting the condition of *Toxochelys latiremis* (Zangerl, 1953a) and *Euclastes wielandi* (Ullmann & Carr, 2021). The hyo-hypoplastron of IRSNB R2 is slightly less ossified than in the juvenile of *Ctenochelys stenopora* described by Matzke (2007) by displaying larger fontanelles but resembles the condition of *Peritresius martini* (Gentry et al., 2018). *Allopleuron hofmanni* also displays squarish fontanelles, but its plastron is significantly less ossified than in IRSNB R2.

The hyo- and hypoplastra present well-developed lateral and medial prongs, indicating that the anterior plastral contacts and the bridge were ligamentous (Fig. 4A–C). The general shape and proportions of the hyo-hypoplastron of IRSNB R2 resemble those of *Peritresius martini* (Gentry et al., 2018), even though these plastral elements are less ossified around the central fontanelle in the latter. On each side, the hyo- and hypoplastra of IRSNB R2 are tightly sutured to one another, making the suture difficult to distinguish in visceral view, although it appears clearly visible on the ventral side of the left hyo-hypoplastron. This difficulty is only increased by the plaster and resin that was used to stabilize the specimen (Fig. 4A, B, D). The medial half of the posterior rim of the hypoplastron presents a deep, obliquely oriented notch for the insertion of the xiphiplastron. The xiphiplastron is narrow, elongated, and oriented anteroposteriorly.

Its medial rim is devoid of prongs but has large, widely spaced, and short rounded serrations, indicating the absence of a median contact with its counterpart. Moreover, the position of the xiphiplastra indicates that a large median fontanelle was probably present between these bones. It is unclear if the xiphiplastron met its counterpart medially at the posterior end of the plastron, as the relevant area is damaged.

#### Plastral scales

Scale sulci are indistinct on the ventral side of the plastron of IRSNB R2 with exception of the left abdomino-femoral and the abdomino-inframarginal scales sulci (Fig. 4A, D, E). The former runs obliquely from the posterior left corner of the central fontanelle to reach the posterior border of the hypoplastron at its maximal concavity whereas the abdomino-inframarginal sulcus diverges from abdomino-femoral sulcus at approximately four centimeters anteriorly to its posterior end, runs obliquely toward the posterior corner of the left lateral fontanelle, to vanish a few centimeters before reaching it.

#### Caudal vertebra

IRSNB R2 preserves a nearly complete caudal vertebra (Fig. 5). The anteroventrally damaged centrum is procoelous, which is the common condition in pan-chelonioids. The posterior facet forms a short, tall, and rounded condyle that displays paired, but damaged posteroventral processes that are possibly remnants of a chevron (Fig. 5B–D). The transverse processes are complete with exception of damage to the right distal end (Fig. 5A, B). The processes are gracile and rod-shaped, as in modern cheloniids (e.g., *Lepidochelys olivacea* QM J85545; *Natator depressus* QM J14463; see also Mulder, 2003) but differ from the short transverse processes of *Dermochelys coriacea* (Gervais, 1872; Völker, 1913). The neural canal is well preserved and roughly circular (Fig. 5A, B). The neural arch is slightly damaged at the anterior confluence of the postzygapophyses (Fig. 5E). A tall, dorsal expansions of the postzygapophysis (epipophyses), as found in *Dermochelys coriacea* (Gervais, 1872), is absent. The neural arch is rather low (Fig. 5), which reminds of *Allopleuron hofmanni* (Mulder, 2003) but contrasts *Dermochelys coriacea* (Gervais, 1872; Völker, 1913) and *Protostega gigas* (USNM 11651 and 11649).

The prezygapophyses are robust, dorsoventrally flattened process with a rounded anterior margin and a convex ventral aspect. In *Protostega gigas*, the prezygapophysis are less rounded, showing a more acute overall shape (USNM 11651 and 11649). The postzygapophyses are short and posterodorsally directed processes with rounded posterior terminations that bear small, flat, and ventrally directed facets (Fig. 5B–D). The posterior terminations of the postzygapophyses do not reach the level of the caudal condyle (Fig. 5C, D), as in *Protostega gigas* (USNM 11651 and 11,649) but contrasting *Allopleuron hofmanni* (IRSNB 3668) in which the postzygapophysis overhang the caudal condyle).

### NHMM 4548

NHMM 4548 preserves 3 adjoining elements of the neural series, two adjoining medial costal fragments (Fig. 6), five more or less complete peripherals of which two are neighboring, a suprapygal, a pygal plate (Fig. 7), as well as a fairly complete plastron (Fig. 8). The original description provided by Kruytzer (1955) indicates that a nearly complete pectoral girdle and more costal fragments were preserved originally, but this material now appears to be missing.

#### Neural series

The neural series of NHMM 4548 is very fragmentary, preserving only three adjoining elements that are associated with two proximal right costal fragments (Fig. 6). The three elements display a tectiform profile due to the presence of a low, wide, and rounded keel (Fig. 6) that is reminiscent of the condition found in the posterior part of IRSNB R2 (Fig. 2A). For simplicity, the three neural elements are referred to as X1, X2, and X3.

The curvature of the intercostal suture (Fig. 6A) indicates that element X1 is the most anterior of the three preserved neurals. The left lateral side of this neural element appears to be damaged. Its right lateral margin is complete and fits with the medial rim of the most posterior costal fragment whereas its posterior side is still articulated with neural element X2. Therefore, it is possible to appreciate that neural element X1 is roughly quadrangular and slightly wider than long. Even though the ventral face of X1 is obstructed by a rounded mass of matrix, the anterior part displays a median ventral keel emerging from the matrix that is compatible with the presence of a neural arch (Fig. 6). It therefore appears likely that X1 represents a genuine neural. The subsequent element designed here as X2 is damaged on the right side, but its left lateral side seems to be nearly complete, and the anterior and posterior margins are in articulation the adjacent neural elements (Fig. 6). X2 is also roughly quadrangular, slightly longer than wide. The low dorsal median keel is crossed by a shallow horizontal groove at the middle of the bony plate that almost cannot be discerned (Fig. 6D, E) and that possibly corresponds to a vertebral scale sulcus track. The visceral face of X2 is damaged and covered by some glue tracks that obscure observation of its morphology. The anterior rim of element X3 is not sufficiently preserved to evaluate its shape. Nevertheless, it appears that element X3 is much wider than the previous ones and its visceral surface is clearly devoid of a contact with a neural arch. So, while it is possible that X3 represents an interneural, the columnar process that posteriorly prolongate the median keel is more reminiscent of those of suprapygal 1 (Fig. 6).

#### Suprapygal and pygal region

NHMM 4548 preserves one isolated element that can be interpreted as a suprapygal 2 (Fig. 7A–D). This element is roughly triangular as preserved, but its broken lateral margins prevent us from being certain of its original shape. The dorsal surface of this element is crossed by a median keel similar, although more marked, to that found on the neural series fragments (Figs. 6, 7A–D). Moreover, its anterior rim has a short, columnar process that is reminiscent of the posterior columnar process of neural element X3 (Figs. 6, 7A–D).

NHMM 4548 also preserves a pygal plate (Fig. 7F–I), but this bone is damaged, lacking its anterior part and its dorsal cortical surface. The lateral sides of the pygal are also missing, making it impossible to appreciate its overall shape. Nevertheless, the pygal of NHMM 4548 reveals the absence of pygal notch, a thickness of around 4 cm, which contrasts with *Euclastes wielandi* (Parris et al., 1986; Ullmann & Carr, 2021), and the presence of ornamentation on the dorsal surface fragment (Fig. 7F). In contrast to NHMM 4548, the presence of a pygal notch is common in pan-chelonioids, as it occurs in ctenochelyids such as *Asmodochelys parhami* (Gentry et al., 2019) and *Prionochelys matutina* (Gentry, 2018) but also in the basal stem chelonioid *Porthochelys laticeps* and *Toxochelys latiremis* as a variable trait (Nicholls, 1988; Zangerl, 1953a), but a notch is absent in the coeval *Allopleuron hofmanni* (Mulder, 2003) and in late Cretaceous protostegids such as *Calcarichelys gemma* (Zangerl, 1953b), *Mesodermochelys undulatus* (Nakajima et al., 2011), and *Protostega gigas* (Zangerl, 1953b). The curvature of the posterior margin is shorter than that of *Allopleuron hofmanni*, which displays an unusual, anchor-shaped, short, and strongly widened pygal plate (Mulder, 2003).

#### Peripherals

The peripherals preserved in NHMM 4548 are fragmentary (Fig. 7J–Y), making it impossible to assess their position with certainty. They all display the ornamental pattern typical of *Glyptochelone suyckerbuykii* on their dorsal and ventral faces and lack the gutter found in *Allopleuron hofmanni* (Mulder, 2003) and *Peritresius martini* (Gentry et al., 2018). Two peripheral fragments display wide angles in cross section suggesting that they are bridge peripherals. One of these is a fragment only (Fig. 7J–M) whereas the second is formed by two adjoining sutured broken peripherals (Fig. 7N–Q). A third fragment is formed by two successive flattened peripherals displaying sections with angles of around 20° and 35°, respectively (Fig. 7R–W), a morphology coherent with the putative isolated peripherals VI and VII of IRSNB R507 (Figs. 9, 10).

#### Plastral bones

The plastron of NHMM 4548 is well preserved, allowing us to appreciate the morphology of the hyo- and hypoplastra as well as the shape of the central and lateral fontanelles (Fig. 8). Nevertheless, some damage is observable to the lateral processes of the right hyoplastron and left hypoplastron, the anterior and medial parts of the left hyoplastron, and the posterior margin of the left hypoplastron. The external surface of the main plates of the hyo- and hypoplastron is well preserved revealing a texturing pattern similar to that found in IRSNB R2 and the presence of some scale sulci (Figs. 4, 8). The right epiplastron is partially preserved with its anterior part missing. The imprint of the posterior process of the entoplastron, as well as some medial digitations of the left hyoplastron, remain visible on the slab (Fig. 8). Both xiphiplastra are missing but the hypo-xiphiplastral suture is preserved on the right side. The size and overall morphology of the plastron of NHMM 4548 is similar to that of the holotype of *Glyptochelone suyckerbuykii* in that both specimens exhibit a large rectangular central fontanelle and small squarish lateral fontanelles, although the central fontanelle of NHMM 4548 is slightly shorter to that of IRSNB R2 (Figs. 4, 8).

The scale sulci and the strongly interdigitated hypo-xiphiplastral contact of NHMM 4548 are similar to those of IRSNB R2 (Figs. 4, 8).

The posterior process of the right epiplastra of NHMM 4548 is thin and elongated and carved by narrow, sagittal ridges but the anterior extremity is sculptured by a few shallow pits that remind of the ornamental pattern found on other surfaces (Fig. 8). The morphology of the posterior part of the epiplastron of NHMM 4548 is similar to that of *Ctenochelys stenopora* (Zangerl, 1953a).

#### Plastral scales

The plastron of NHMM 4548 preserves more scales sulci than IRSNB R2, in particular the pectoro-abdominal, abdomino-femoral, and abdomino-inframarginal sulci. This confirms the universal presence of plastral scales in *Glyptochelone suyckerbuykii* (Figs. 4, 8). The pectoro-abdominal sulcus of NHMM 4548 is nearly horizontal and spread from the anterior medial corner of the lateral plastral fontanelles to the anterior lateral corner of the central plastral fontanelle. The scale sulci pattern exhibited by NHMM 4548 and IRSNB R2 is coherent with the condition of modern cheloniids (Hutchison & Bramble, 1981). Therefore, the preserved inframarginal likely represents inframarginal IV.

### IRSNB R507

IRSNB R507 consists of a disarticulated shell that includes a fairly complete carapace, some plastral fragments, a partial pelvic girdle, as well as a proximal left femur (Figs. 9, 10, 11, 12, 13). The shell is covered by a surface ornamentation highly reminiscent of IRSNB R2 and NHMM 4548 (Figs. 2A, C, 7E, 9C, 15A, C), consisting of crests and vermiculations radiating from ossification centers of the bony plates and distinct, rounded foramina. The neural series of IRSNB R507 preserves nine neural/interneural elements (Figs. 9A, C, 10A, B). The left costals I to VII and the right costals I to VIII are preserved, though most of them are incomplete. A partial peripheral ring is present, consisting of the second to fourth left peripherals, as well as three isolated, more posterior peripherals. Although their exact positions are uncertain, we tentatively identify them as the left 6th and the right 7th and 8th peripherals (Fig. 9A, B) based on their cross-sectional morphology and the position of their rib insertion pits (see below). The carapacial remains suggest that the overall shape of the shell of IRSNB R507 was nearly rounded (Fig. 9A, B), reminiscent of the condition found in *Euclastes wielandi* (Ullmann & Carr, 2021), slightly more elongated than *Toxochelys moorevillensis* (Zangerl, 1953a), but in strong contrast to the holotype of *Glyptochelone suyckerbuykii* IRSNB R2 (Fig. 2A, C). The plastron consists of the nearly complete left xiphiplastron, a fragment of the right xiphiplastron, and fragments of the right hyoplastron and hypoplastron.

#### Nuchal region

The nuchal bone is not preserved in IRSNB R507, but the postnuchal fontanelles typical of ctenochelyids and *Toxochelys* spp. (Baird, 1964; Zangerl, 1953a) were possibly present in IRSNB R507, as indicated by the shape of the anteromedial edge of its left first costal, which displays a rounded excavation (Fig. 9A, B). Although the margins of the costals do not seem to show any traces of breakages, the asymmetrical appearance of these “fontanelles” nonetheless leads us to remain cautious about their nature.

#### Preneural

As in IRSNB R2, no preneural element is preserved in IRSNB R507, but as the first neural of IRSNB R507 overhangs both the second and first thoracic vertebrae presence of a preneural appears unlikely (Fig. 10B).

#### Neural series

Epineural ossifications and a median keel are absent in IRSNB R507 (Fig. 9A, B), reminding of IRSNB R2. The neural series of IRSNB R507 preserves nine neural/interneural elements, but space is available between the eighth costals for a tenth or possibly even an eleventh neural/interneural element (alternative: element of the neural series). The shape of the posterior elements of the neural series as well as the absence of neural arches on elements 6 and 8 indicate that the latter bones are interneurals 5/6 and 6/7. These are broad and hexagonal plates followed by the small and squarish neurals VI and VII (see Figs. 9A–B, 10A, C). Therefore, the neural formula of IRSNB R507 is 4, 6 A, 6 A, 6 A, 6 A, I6P, 4, I6P, 4,?.

Neural I is narrow, elongated, and quadrangular in shape (Figs. 9, 10). Neurals II to IV are hexagonal with short anterior sides (6A of Pritchard, 1988). They show short anterolateral contacts with costals I to IV and long posterolateral contacts with costals II to V.

IRSNB R507 includes an irregular, supernumerary element that is located at the left anterolateral corner of neural element IV (Fig. 9A, B). It is noteworthy that both textured turtles from the latest Cretaceous of the Belgium and Netherlands (IRSNB R2 and R507) display abnormal elements in their neural series (Figs. 2A–B, 9A–B; see Discussion).

The neurals VI and VII of IRSNB R507 are preceded by hexagonal interneural elements, interneurals 5/6 and 6/7, that are wider than long. These elements lack a contact with the thoracic vertebrae and therefore do not represent true neurals (i.e., neural arch expansions). The neurals VI and VII of IRSNB R507 are small, rounded, and solidly connected along their visceral faces to the neural arches of thoracic vertebrae VII and VIII (Figs. 9, 10). The dorsal surfaces of the posterior neurals elements are extremely flat, which likely indicates the absence of the tectiform shape found in IRSNB R2 and NHMM 4548 (see above). However, due to the absence of the eight neural, an eventual postneural, the suprapygals, and the pygal, we remain cautious about this difference.

In visceral view (Fig. 10A, C), IRSNB R507 preserves some parts of the thoracic vertebral series, but most of the centra are missing. The visceral face of the first neural bears the neural arch of the second thoracic vertebra (Fig. 10). As is typical in turtles, this first thoracic vertebra is shorter than the adjacent thoracic vertebrae. The dorsal facet of the prezygapophyses of the first thoracic vertebra of IRSNB R507 (Fig. 10B) is horizontally oriented, contrasting with the condition found in the type specimen of *Glyptochelone suyckerbuykii* (IRSNB R2, see above), where the prezygapophyses are slightly descending anteriorly. In modern cheloniids, the first thoracic prezygapophyses are mostly oriented horizontally but still slightly descending anteriorly, as in IRSNB R2. Unfortunately, the anterior articulation facet with the eight cervical is not preserved in IRSNB R507 with the remainder of the centrum. The only other centrum that is partially preserved in IRSNB R507 is that of the sixth thoracic vertebrae, which is still articulated with neural V. This centrum is devoid of a keel and presents a slight narrowing in its central part, a condition similar to that of the type specimen of *Glyptochelone suyckerbuykii* (IRSNB R2, see above). Neurals VI and VII of IRSNB R507 display no preserved remains of vertebral centrum on the visceral face but partial neural arches are preserved on their visceral surface (Fig. 9A, C).

#### Costals

The costal plates of IRSNB R507 (Figs. 9, 10) are quite thin, around 4 mm thick at their anterior and posterior margins. Most costal plates are at least partially preserved, even though most of the free rib ends are broken or missing. The only fully missing costal plate is the left eighth one. The free rib of the left costal V (Figs. 9, 10) is complete and still in articulation, allowing us to appreciate that the length of the costal callosities in IRSNB R507 is slightly shorter than the length of the free rib, an ossification degree similar to that of *Prionochelys matutina* (Gentry, 2018), less ossified than the type of *Glyptochelone suyckerbuykii* (IRSNB R2, see above), *Ctenochelys stenopora* (see type specimen of *Toxochelys bauri*, YPM 1786 in Wieland, 1905), and *Toxochelys moorevillensis* (Zangerl, 1953a), but more ossified than *Allopleuron hofmanni* (Mulder, 2003). Additionally, the ribs of IRSNB R507 (Figs. 9, 10) emerge progressively at the lateral edges of the costal plates, resembling the condition seen in *Prionochelys matutina* (Gentry, 2018), *Ctenochelys stenopora* (see type specimen of *Toxochelys bauri*, YPM 1786 in Wieland, 1905), and *Euclastes wielandi* (Ullmann & Carr, 2021), but contrasting with the condition found in *Allopleuron hofmanni* (Mulder, 2003). Consequently, the costoperipheral fontanelles of IRSNB R507 appear to have a elliptic shape. The type of *Glyptochelone suyckerbuykii* IRSNB R2 (Fig. 2A, B) displays an intermediate condition between IRSNB R507 and *Allopleuron hofmanni*, with free rib ends that emerge progressively from the ribs but bordered by abrupt, vertically oriented anterior and posterior rims. The visceral view of IRSNB R507 (Fig. 10) preserves some rib heads, of which the second right, the fourth right, and both fifth rib heads are complete and show a triangular shape. The second right rib head has two flat medial facets, an anterior one that was articulated with the second thoracic vertebra and a more posterior one that was in contact with the third thoracic vertebra. The other preserved rib heads have only one articular facet that is more rounded. The first rib of IRSNB R507 is not preserved, but a bulge is present anterior to the merging between the second rib and the first costal plate, indicating that a vestigial first rib was present and merged with the second rib, as in most sea turtles. The costal plates of IRSNB R507 display an unusually smooth and flat visceral surface (Fig. 10A), making the ribs indistinct lateral to their merging point with the costal plate. This condition resembles what is known in the type specimen of *Glyptochelone suyckerbuykii* (IRSNB R2, see above), where the ribs are difficult to discern in the visceral view of costals. This condition contrasts with many Cretaceous pan-chelonioids such as *Euclastes wielandi* (NJSM 12295), *Ctenochelys stenopora* (Matzke, 2007), *Toxochelys latiremis* (Nicholls, 1988), *Allopleuron hofmanni* (Mulder, 2003) but also differs from modern cheloniids (Zangerl & Turnbull, 1955; Additional file 8, Fig. 2). Moreover, the visceral face of the first costal plates of IRSNB R507 (Fig. 10A) is very flat at their medial third, suggesting the absence of the deep pocket found in the type *Glyptochelone suyckerbuykii* (IRSNB R2, Fig. 3A). However, in the absence of the nuchal bone in IRSNB R507, it is impossible to be certain about this character.

#### Peripherals

IRSNB R507 preserves a partial peripheral ring consisting of left peripherals II to IV and three isolated, nearly complete peripheral elements (Figs. 9A–B, 10A, C). It is noteworthy that IRSNB R507 is incorrectly mounted. Only the left peripherals II to IV are correctly positioned in the armature (digitally removed from the figured), whereas the three more posterior ones have odd positions. Two “peripheral X” elements display significantly different morphologies with completely different cross-sectional shapes, and an odd “peripheral XI” displays an unusually wide angle in cross-section. We provide a new assessment for the peripheral positions based on the morphology of their cross-sections and comparison with those of IRSNB R2, which tend to display lower angles posteriorly, as well as the medial faces that bear the insertion pits and a groove that generally disappears posteriorly (Fig. 9A). Moreover, the rib insertion pits of the posterior peripherals, where present, tend to be positioned at the posterior half of the plate. Therefore, we tentatively repositioned the “right peripheral XI” to a more realistic position as left VI, the “right X” as right VII, and the “left X” as left peripheral VIII (Figs. 9A–B, 10A–C). IRSNB R507 shares with the type specimen of *Glyptochelone suyckerbuykii* (IRSNB R2, see above) the absence of serrations on its peripherals, in contrast not only with ctenochelyids (Figs. 2A–B, 9A–B, 10A–C; e.g., Gentry, 2018; Gentry et al., 2018) but also *Allopleuron hofmanni* (Mulder, 2003), the absence of the peripheral gutter found in *Allopleuron hofmanni* (Mulder, 2003), as well as the presence of the ornamental pattern, although shallow, on the dorsal and ventral faces of the plates. The second peripheral is nearly complete and presents a “banana-shaped” anterior cross-section (Fig. 9A), is more elongated, but otherwise resembles that of the type specimen of *Glyptochelone suyckerbuykii* (IRSNB R2). The posterior cross-section of the second peripheral of IRSNB R507 is “drop-shaped,” and its lateral walls form an angle of around 35°. The second peripheral is devoid of a trough and rib insertion pit, indicating that it represents an anterior free rib peripheral. At the level of the third peripheral, the angle formed by the two lateral walls abruptly increases to reach an angle of about 95° (Fig. 9A) and the medial face presents a wide medial trough carved by a broad rib insertion pit centered at the end of the anterior third of the bone. The fourth peripheral of IRSNB R507 is damaged at its midpoint, corresponding to the location of the rib insertion pit that must lie in a wide medial trough. The anterior and posterior cross-sections of the fourth peripheral of IRSNB R507, respectively, display angles between the lateral walls of around 90° and 70°.

The “right peripheral XI” is tentatively positioned to a more realistic position as left VI because the anterior and posterior angles are at about 50 and 35°, respectively, and because the position of the insertion pit, situated at the posterior margin of the second third of the bone corresponds to what is found in R2 for the peripheral VI. This assessment needs to be moderated by the fact that both peripherals VI of IRSNB R2 are damaged (Fig. 2 A, C, Additional file 8).

The right “peripheral X” displays a morphology surprisingly similar to the peripheral VII of the holotype of *Glyptochelone suyckerbuykii* IRSNB R2 with an anterior angle of 35° and a posterior angle of 25° as well as a deep and narrow trough with a distinct insertion pit that is situated at the posterior third of the bone (Figs. 2A, 9A; Additional file 8). It is here therefore inferred to be a left VII peripheral. We are more confident about this assessment than that of the peripheral VI.

The position of the “left peripheral X” is more uncertain. Indeed, its extremely flattened morphology that displays anterior and posterior angles of approximately 25° and 15°, respectively, does not correspond to any peripherals preserved in IRSNB R2 (Fig. 2A, C; Additional file 8) and lead us to place it more posteriorly. Moreover, its anterior angle around 25° is similar of the posterior angle known for the putative seventh peripheral and its trough is shallow, strongly narrowed and displays a shallow insertion pit at its posterior third. This might suggest that it is an left eight peripheral, but it remains plausible that it constitutes a left nineth of tenth peripheral.

If the reconstruction is correct, IRSNB R507 displays a flattening of the posterior peripherals that reminds the condition of *Euclastes*-like sea turtles and differs from the condition of *Allopleuron hofmanni* in which the posterior peripherals become flatter but remain “drop-shaped” with an angle about 30° (Mulder, 2003).

#### Plastral bones

The plastron of IRSNB R507 is fragmentary (Fig. 11). The putative right hyoplastron is reduced to a fragment, but the right hypoplastron shows presence of ornamentation, including foramina (Fig. 11D–F). The xiphiplastra of IRSNB R507 are notably devoid of deep ornamentation, in contrast with the holotype of *Glyptochelone suyckerbuykii* (compare Fig. 4A, C with Fig. 11H–J). The left xiphiplastron of IRSNB R507 preserves a part of its contact with the hypoplastron, consisting of a deep pointed notch into which the latter made an incursion (Fig. 11I, J). Overall, the plastral morphology is coherent with the holotype IRSNB R2 (Figs. 4, 11).

#### Scale sulci

As in the holotype of *Glyptochelone suyckerbuykii* (IRSNB R2, see above), most of the marginal sulci are not visible in IRSNB R507 (Fig. 9A, B). The only marginal sulci visible run on the dorsal surface of the second left peripheral as well as on the ventral face of the seventh right peripheral. Only the posterior left pleural I can be traced on the second left costal plate (Fig. 9A, B).

The vertebral sulci, by contrast, are deeply marked on the surface of the costals and neurals. The neurals I, III, and V are crossed by the posterior sulcus of vertebral scales I, II, and III, respectively (Fig. 9A, B). The posterior sulcus of vertebral IV is visible on the right costal VIII, runs posteriorly to neural VII, and likely cross neural VIII, which is absent. Vertebrals II and III of IRSNB R507 are clearly wider than long and display well-marked “pinched” lateral extensions, probably at the junction of the pleural scales, which cover the dorsal surface of costals II and IV, respectively (Fig. 9A, B). This condition differ drastically from what is found in the type specimen of *Glyptochelone suyckerbuykii* IRSNB R2 (compare Fig. 2A, B with Fig. 9A, B; see above), which presents vertebrals II to IV that are significantly longer than wide and only have small “pinched” lateral extensions. Moreover, the vertebrals II and III of IRSNB R507 occupy a wider space on the costals, reaching the lateralmost third at their maximal width, whereas the same scales are restricted to the medialmost third of the costals in the type specimen of *Glyptochelone suyckerbuykii*. Nevertheless, these differences can be explained by ontogenetic variation (see Discussion). In addition, the shape of IRSNB R507 corresponds to a vertically compressed version of what is found in the type specimen, displaying also hexagonal vertebrals II and III. However, the fourth vertebral of IRSNB R507 is slightly wider than long and displays an irregular form reminiscent of the unusual octagonal shape of this element in IRSNB R2. In *Allopleuron hofmanni*, the vertebral scales, when visible, are extremely narrow, limited to the neural series (Mulder, 2003), or only barely reach the most medial parts of the costals (see IRSNB R8). In *Ctenochelys stenopora*, the vertebral series is narrower than that found in IRSNB R507, resembling the condition of the type specimen of *Glyptochelone suyckerbuykii* (Wieland, 1905) even at a juvenile stage (Matzke, 2007). In addition, the fourth vertebral of *Ctenochelys stenopora* displays an elongated heptagonal shape (Matzke, 2007) with short anterolateral sides, contrasting with both *Glyptochelone suyckerbuykii* specimens (Figs. 2A–B, 9A, B). A narrow vertebral series is also present in *Peritresius ornatus*, with proportions resembling those of the type of *Glyptochelone suyckerbuykii* (IRSNB R2), but the vertebrals II and III of *Peritresius ornatus* display a quadratic shape and its vertebral IV is pentagonal, contrasting with the former (Baird, 1964). In *Euclastes wielandi*, the vertebral series is wider than long (Parris et al., 1986; Ullmann & Carr, 2021), resembling the condition found in IRSNB R507 (even if the vertebral IV of NJSM 12295 is slightly longer than wide, Parris et al., 1986), but the vertebral IV is otherwise hexagonal, contrasting with both *Glyptochelone suyckerbuykii* specimens (Figs. 2A–B, 9A, B).

#### Pelvic girdle

The pelvic girdle of IRSNB R507 includes the nearly complete pubes and ilia (Fig. 12). The pubis and ilium display similar lengths, resembling the condition found in ctenochelyids such as *Ctenochelys stenopora* (Matzke, 2007; Zangerl, 1953a), *Peritresius martini* (Gentry et al., 2018), *Prionochelys matutina* (Gentry, 2018), and *Toxochelys moorevillensis* (Zangerl, 1953a). This condition contrasts with chelydrids, in which the ilium is much longer than the pubis, as well as modern cheloniids, in which the ilium is shorter than the pubis (Zangerl, 1953a).

Both pubes of IRSNB R507 (Fig. 12) are nearly complete, but the median epipubic process is not preserved, suggesting that it was likely not ossified, contrasting with *Peritresius ornatus* (Gentry et al., 2018), *Dermochelys coriacea*, *Eosphargis gigas* (Seago, 1979), *Erquelinnesia gosseleti* (Zangerl, 1971), and *Allopleuron hofmanni* (Mulder, 2003). The pubic blade is well-developed. Its anterior point reaches the level of the pectineal process. The pubes of IRSNB R507 display a robust, laterally directed pectineal process that is nearly quadratic and finished by a broad, smooth, rounded “point” (Fig. 12). The pectineal process of IRSNB R507 is anteriorly bordered by a wide emargination, resembling the condition found in *Allopleuron hofmanni* (Mulder, 2003), but contrasting with the ctenochelyid *Peritresius martini* (Gentry et al., 2018) and *Erquelinnesia gosseleti* (Zangerl, 1971), which display a much narrower and deeper emargination at its anterior border. *Dermochelys coriacea* displays a much-elongated pectineal process than IRSNB R507, anteriorly bordered by a deeper anterior notch (IRSNB 5107; Seago, 1979). The acetabulum of IRSNB R507 is directed ventrally, as in ctenochelyids (Gentry, 2018; Gentry et al., 2018). The iliac facet of the pubis of IRSNB R507 (Fig. 12) is short and strongly laterally oriented, contrasting with *Ctenochelys stenopora* (Matzke, 2007) and *Prionochelys matutina* (Gentry, 2018). This resembles what is known in *Erquelinnesia gosseleti*, where the iliac facet is also more laterally oriented (Zangerl, 1971), even though in the latter, the facet is longer.

Both ilia of IRSNB R507 (Fig. 12) display very smooth dorsal and ventral surfaces and are devoid of the lateral tuberosity found in *Peritresius martini* (Gentry et al., 2018). The iliac neck of IRSNB R507 is more elongated than that found in ctenochelyids (Gentry, 2018; Gentry et al., 2018; Matzke, 2007), resembling the proportions of *Allopleuron hofmanni* (Mulder, 2003). Nevertheless, the ilium of IRSNB R507 (Fig. 12) contrasts with the latter by its smooth, nearly cylindrical neck that is devoid of ridges and grooves found, in particular, on the dorsal surface of *Allopleuron hofmanni* (Mulder, 2003). Both iliac blades of IRSNB R507 (Fig. 12) are incomplete, making it difficult to evaluate their total length. Nevertheless, this structure is sufficiently preserved to observe that the angle formed by the iliac blade with the iliac neck is wider than that found in many Cretaceous pan-chelonioids, including *Toxochelys moorevillensis* (Zangerl, 1953a), *Ctenochelys stenopora* (FMNH PR248), and *Erquelinnesia gosseleti* (Zangerl, 1971). This character seems to be variable in *Toxochelys latiremis*, with some specimens displaying an iliac neck-blade angle similar to those of IRSNB R507 (see YPM 1385), whereas others show a significantly smaller angle (YPM 3602). In *Dermochelys coriacea* (Seago, 1979) and in modern cheloniids such as *Natator depressus* (Zangerl, 1988) and *Eretmochelys imbricata* (Zangerl, 1953a), the iliac blade is reduced, and therefore, the ilium consists only of a rod-like bone. The overall shape of the ilium of IRSNB R507 resembles that of *Chelydra serpentina*.

#### Femur

The left femur of IRSNB R507 fairly complete, lacking parts of the femoral head and the lateral part of the minor trochanter (Fig. 13). The femur of IRSNB R507 exhibits a flat and high major trochanter, a well-marked intertrochanteric fossa, and a minor trochanter that is distinct and positioned slightly more distally than the femoral head. In *Toxochelys moorevillensis*, the major trochanter is reduced and fused with the femoral head (Zangerl, 1953a), contrasting with the condition displayed by IRSNB R507 (Fig. 13). In most protostegids (Elliott, 1997; Hirayama & Chitoku, 1996; Lehman & Tomlinson, 2004; Wieland, 1902; Zangerl & Sloan, 1960), the intertrochanteric fossa is shallow, and the major trochanter is clearly lower than the femoral head.

In dermochelyids (Nielsen, 1964; *Dermochelys coriacea* QMJ47453), the intertrochanteric fossa is reduced in size, as well as the major trochanter, which forms a low bulge instead of the flat and high crest found in IRSNB R507. *Allopleuron hofmanni* also displays reduced trochanters, but its femoral head is more lateralized (Mulder, 2003). Since most femora known from *Allopleuron hofmanni* are still articulated or embedded in matrix, no specimens displaying the intertrochanteric fossa are available for this taxon. In modern cheloniids, the femur differs greatly from IRSNB R507 (Fig. 13) as the major trochanter is lower, around the height or below the femoral head, forming a wide bulge, and the intertrochanteric fossa is much smaller and shallower (Evers et al., 2019; Koolstra et al., 2019, supplements). Although some extinct cheloniids display a much more elevated major trochanter, such as “*Syllomus aegyptiacus*” (Hasegawa et al., 2005; Weems, 1974) and *Eochelone brabantica* (see Evers et al., 2019, supplements), the latter displays a morphology that resembles IRSNB R507 (Fig. 13).

The overall morphology of the femur of IRSNB R507 (Fig. 13) including the horizontal medial orientation of the femoral head, the crest-like major trochanter, and the rectangular minor trochanter in anterior view separated by deep ridges reminds of thalassochelydians such as *Jurassichelon oleronensis* (see Evers et al., 2019, supplement) and *Neusticemys neuquina* (de la Fuente & Fernandez, 2011). The femur is similar to that of *Ctenochelys* spp. (see *Ctenochelys tenuitesta* FMNH P27563 in Zangerl, 1953a) by displaying a laminar shaped major trochanter, as well as marked intertrochanteric fossae, and a well separated minor trochanter.

## Phylogeny

Our preferred phylogenetic analysis, which was performed using implied weighting (k = 12) and ordered morphoclines (see Materials and Methods above), resulted in 4 MPTs that imply 1786 character state transitions (Additional file 4).

**Fig. 14:**
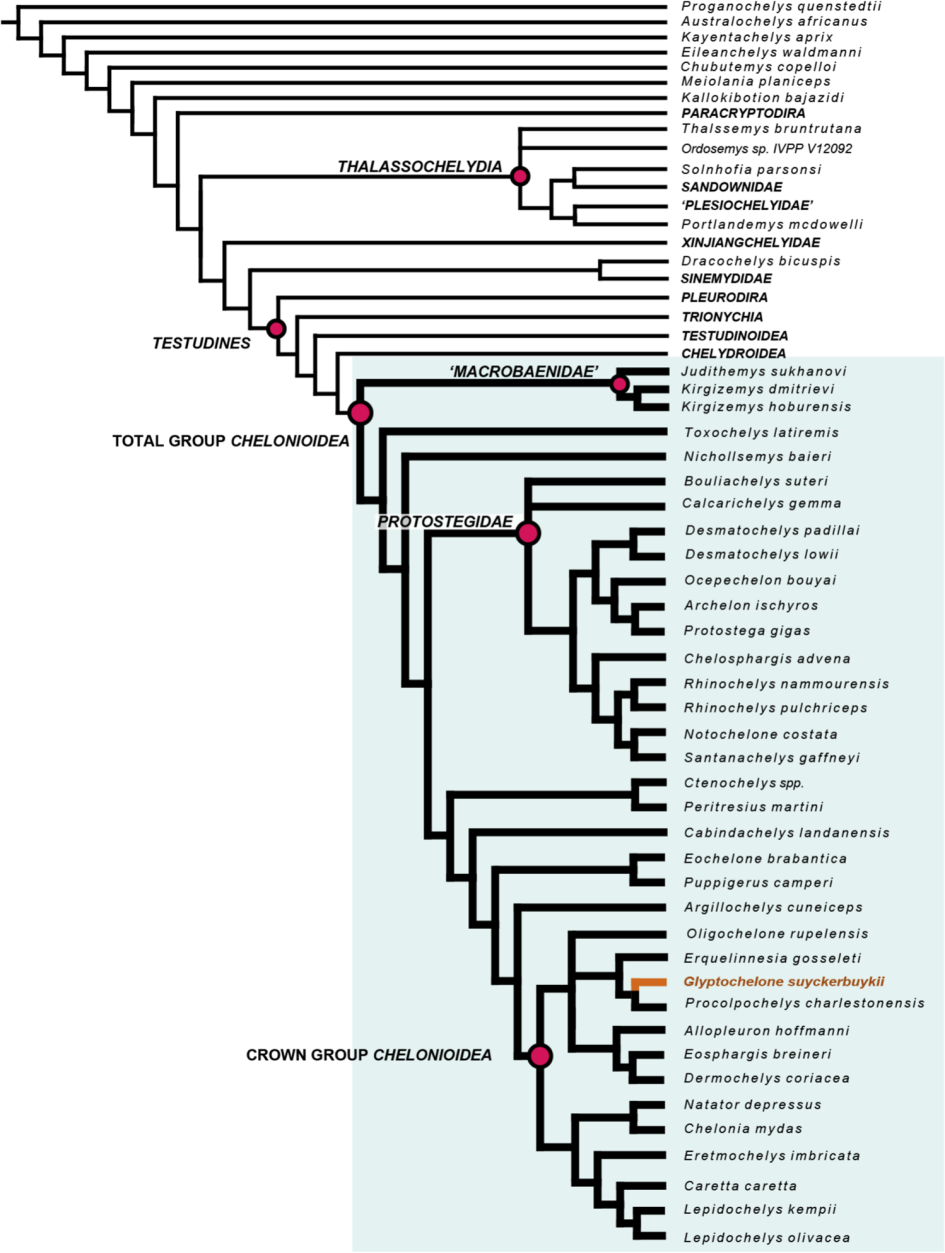
Strict consensus tree obtained with the MPTs from the parsimony analysis run with implied weighting (k = 12). All the resulting MPTs are available in Additional file 4

*Glyptochelone suyckerbuykii* is hypothesized to be placed inside crown group *Chelonioidea*, retrieved as sister to *Procolpochelys charlestonensis* within *Dermochelyidae* (Fig. 14). The same placement is retrieved in our exploratory analysis performed without implied weighting (see strict consensus of 720 MPTs in Additional file 3).

The hypothesized content of *Dermochelyidae*, similar to that found by Menon et al. (2024), appears dubious, by including the putative stem-cheloniids *Procolpochelys charlestonensis* and *Oligochelone rupeliensis* and the *Euclastes*-like durophagous chelonioid *Erquelinnesia gosseleti*, in addition to the plausible stem-dermochelyid *Allopleuron hofmanni* and the universally accepted dermochelyid *Eosphargis breineri*. One explanation could be the grouping of these taxa by homoplastic features, as most “synapomorphies” for this group are otherwise highly homoplastic, including the absence of the vertical flange on the external pterygoid process (ch.102:0, ACCTRAN), absence of a symphyseal ridge on the dentary (ch.176:0; DELTRAN), and the full enclosure of the ectepicondylar opening into a canal rather than a groove (ch.330:0; unambiguous). Alternatively, this unusual grouping could be the result of the meager morphological overlap between taxa, as some of these synapomorphies are not known for some of the affected taxa. For example, the status of the vertical flange on the external pterygoid process (ch.102:0) is unknown in *Oligochelone rupeliensis* and *Glyptochelone suyckerbuykii* because cranial and mandibular remains are missing that could contradict their placement inside *Dermochelyidae*. In addition, although the available remains are fairly complete (Weems & Brown, 2017), *Procolpochelys charlestonensis* is poorly described resulting in incomplete scorings for this taxon. A possibility to resolve this problem could be the inclusion of additional taxa of putative fossil pan-cheloniids, such as *Carolinochelys wilsoni* (Hay, 1923; Weems & Sanders, 2014) or *Trachyaspis lardyi* (e.g., Hasegawa et al., 2005; Meyer, 1843; Villa & Raineri, 2015) but also more plesiomorphic representatives of *Euclastes*-like chelonioids, such as *Euclastes wielandi* (Ullmann & Carr, 2021) and *Mexichelys coahuilaensis* (Brinkman et al., 2009).

In an attempt to explore the effect of alternative topologies on tree length, we moved *Glyptochelone suyckerbuykii* into more basal positions using Mesquite’s Move Branch tool in the Tree Info Panel (Additional file 8, Figs. 5, 6). The placement of *Glyptochelone suyckerbuykii* at the basis of dermochelyids resulted in an increase of tree length of only two steps. All other alternative positions for this taxon resulted in an increase of tree length by at least 4 more steps. However, the joint displacement of the clade (*Erquelinnesia* (*Glyptochelone* + *Procolpochelys*)) basal to cheloniids or basal to the chelonioid-crown group only increased tree length by one step. This result leads us to be particularly cautious about the placement of this group within dermochelyids.

The clade *Glyptochelone suyckerbuykii* + *Procolpochelys charlestonensis* is only united by the presence of interneurals (ch.206:2, unambiguous) and presence of two or more interneurals (ch.207:2, DELTRAN). As these two taxa are only united by character related to the interneurals, we expect this result to be tested by a more detailed description of *Procolpochelys charlestonensis*. Incidentally, presence of two or more interneurals is also retrieved as an ACCTRAN synapomorphy for the dermochelyid group, even if it is absent in *Allopleuron hofmanni, Eosphargis breineri* and *Dermochelys coriacea*. This further highlights issues associated with this purported clade.

*Glyptochelone suyckerbuykii* is characterized by three autapomorphies of which two are unambiguous, one only in DELTRAN, in particular the presence of a texturing pattern (ch.192:1), the loss of a raised nuchal pedestal (ch.196:0, DELTRAN), and the presence of a deep notch separating the femoral head and the major trochanter (ch.357:0). We note that the absence of a nuchal pedestal was retrieved in ACCTRAN as a synapomorphy for the dermochelyid but reverses to present in *Dermochelys coriacea*.

The overall topology (Fig. 14) and the synapomorphies that support various clades are similar to those found in Menon et al. (2024; see Additional file 6 for more details). We note the following additional points of interest: (1) *Allopleuron hofmanni* is placed as a dermochelyid just outside the clade of *Eosphargis breineri* and *Dermochelys coriacea* (as in Gentry et al., 2019). (2) *Argillochelys cuneiceps*, *Puppigerus camperi, and Eochelone brabantica* are found as stem-chelonioids in a node more crownward than ctenochelyids, not as stem-cheloniids (e.g., Evers & Benson, 2019; Gentry et al., 2019). (3) Ctenochelyids are retrieved as stem chelonioids (as is Gentry, 2018; Gentry et al., 2019) one node more crownwardly than protostegids and not as stem-cheloniids (e.g., Gentry et al., 2018; Joyce et al., 2021a). (4) Protostegids are found to be stem chelonioids in a more crownward position than *Nichollsemys baieri*. (5) *Toxochelys latiremis* is placed one step more stemward than *Nichollsemys baieri* instead of as sister to this taxon (as found in Brinkman et al., 2006 and Menon et al., 2024). (6) Finally, some “macrobaenids” (*Kirgizemys* spp., *Judithemys sukhanovi*) are found as the earliest-branching stem chelonioids (e.g., Cadena and Parham, 2015; Joyce et al., 2021a; Menon et al., 2024).

When a concavity constant of k = 7 is implemented, following the recommendations of Escurra et al. (2024), *Sandownidae* is retrieved within *Pan-Chelonioidea* one step more crownward than a clade formed by *Judithemys sukhanovi* and *Kirgizemys* spp. (Additional file 3). When using equal weights, *Thalassochelydia* is retrieved in this position (Additional file 5; see Additional file 7 for a complete list of synapomorphies). As these results are stratigraphically implausible and contradict most recent studies (Evers & Benson, 2019; Evers et al., 2019; Gentry, 2018; Gentry et al., 2018; Joyce et al., 2021b) but were already retrieved by Gentry et al. (2019)), we give preference to the analysis using a concavity constant of k = 12.

## Discussion

### Alpha taxonomy

We here redescribe three Late Cretaceous fossil turtle specimens that are united by the presence of a distinct, textured shell surface pattern, one of which is the holotype of *Glyptochelone suyckerbuykii* (Ubaghs, 1879). Even though all three specimens were collected near the Belgian-Dutch border, they originate from two different formations separated by an important temporal hiatus: IRSNB R2, the holotype, and NHMM 4548 originate from the late Maastrichtian Mersen Member of the Maastricht Formation, while IRSNB R507 was collected from the late Campanian Spiennes Chalk. So, while the referral of NHMM 4548 to *Glyptochelone suyckerbuykii* is supported by morphological and temporal arguments, the referral of IRSNB R507 based on texture pattern alone appears insufficient.

IRSNB R2 and IRSNB R507 displays a number of anatomical differences that initially lead us to be skeptical about the identification of IRSNB R507 as representative of *Glyptochelone suyckerbuykii*: (1) the shell of IRSNB R507 is more rounded; (2) vertebrals II and IV are proportionally much wider in IRSNB R507 than in IRSNB R2; (3) postnuchal fontanelles were possibly present in IRSNB R507, but certainly not in IRSNB R2; (4) costoperipheral fontanelles are more elongated and the rib ends emerge more progressively from the costals in IRSNB R507 than in IRSNB R2; (5) only neurals VI and VII are preceded by interneurals in IRSNB R507 whereas interneurals occurs four times between neurals II–III, III–IV, IV–V, and V–VI in IRSNB R2; and (6) the xiphiplastral ornamentation pattern is shallower in IRSNB R507 than in IRSNB R2.

The significance of the above listed differences is reduced by the observation that IRSNB R507 is significantly smaller than IRSNB R2, the holotype of *Glyptochelone suyckerbuykii*, and may therefore represent a younger ontogenetic stage. Indeed, previous studies on extant cheloniids (Salmon & Scholl, 2014; Salmon et al., 2015) have shown that younger individuals tend to be more rounded, possibly as a defense against gape limited predators (Salmon & Scholl, 2014; Salmon et al., 2015). Among fossil marine turtles, this variation is also known in *Ctenochelys stenoporus* (compare the holotype of *Toxochelys bauri* in Wieland, 1905 and the juvenile in Matzke, 2007) but these differences seem to be absent in *Dermochelys coriacea* (Pate & Salmon, 2017). Similarly, while postnuchal fontanelles occur in subadult extant chelonioids, these openings tend to be reduced in adult forms. A similar trend is also apparent for the vertebral scales, which have a tendency to become more elongated in the adults of modern cheloniids (Salmon et al., 2015). The costoperipheral fontanelles also have a tendency to reduce across ontogeny due to the progressive ossification of the shell (Cherepanov, 2019; Kordikova, 2002). Finally, the more progressive emergence of the rib ends can be also explained by ontogeny because the shell becomes more elongated in more adult forms, which means that the late phase of growth focuses on the anteroposterior elongation for the costals generating straighter lateral rims at their anterior and posterior extremities (Additional file 8). Although variation to the positions and number of interneurals within the neural series cannot be explained by ontogenetic variation, this character is known to be highly variable in current cheloniids, in particular in *Lepidochelys* spp. (Kordikova, 2002; Pritchard, 1988; Zangerl & Turnbull, 1955) and *Natator depressus* (Pritchard, 1988; Zangerl, 1988). The great amount of variation apparent to the exact texture pattern of other groups of turtles (see Joyce, 2025a for trionychids) suggests to us that the minor differences in texture pattern between IRSNB R507 and IRNSB R2 should not be overinterpreted. Indeed, in addition to the unusual shell texturing pattern, both IRSNB R2 and R507 shares an unusual octagonal shaped vertebral scale IV and the presence of interneurals, characters unique among Late Cretaceous sea turtles.

In conclusion, it appears that all differences observed between IRSNB R507 and IRNSB R2, the holotype of *Glyptochelone suyckerbuykii*, are best interpreted as the result of a combination of ontogenetic and intraspecific variation. This leads us, for now, to consider them as belonging to the same species. Nevertheless, the long hiatus of around 10 million years that separate these two specimens, despite their geographical proximity, (Fig. 1), increases the chances that further discoveries, particularly cranial remains, will contradict this assessment.

### Shell surface ornamentation in Late Cretaceous sea turtles

**Fig. 15:**
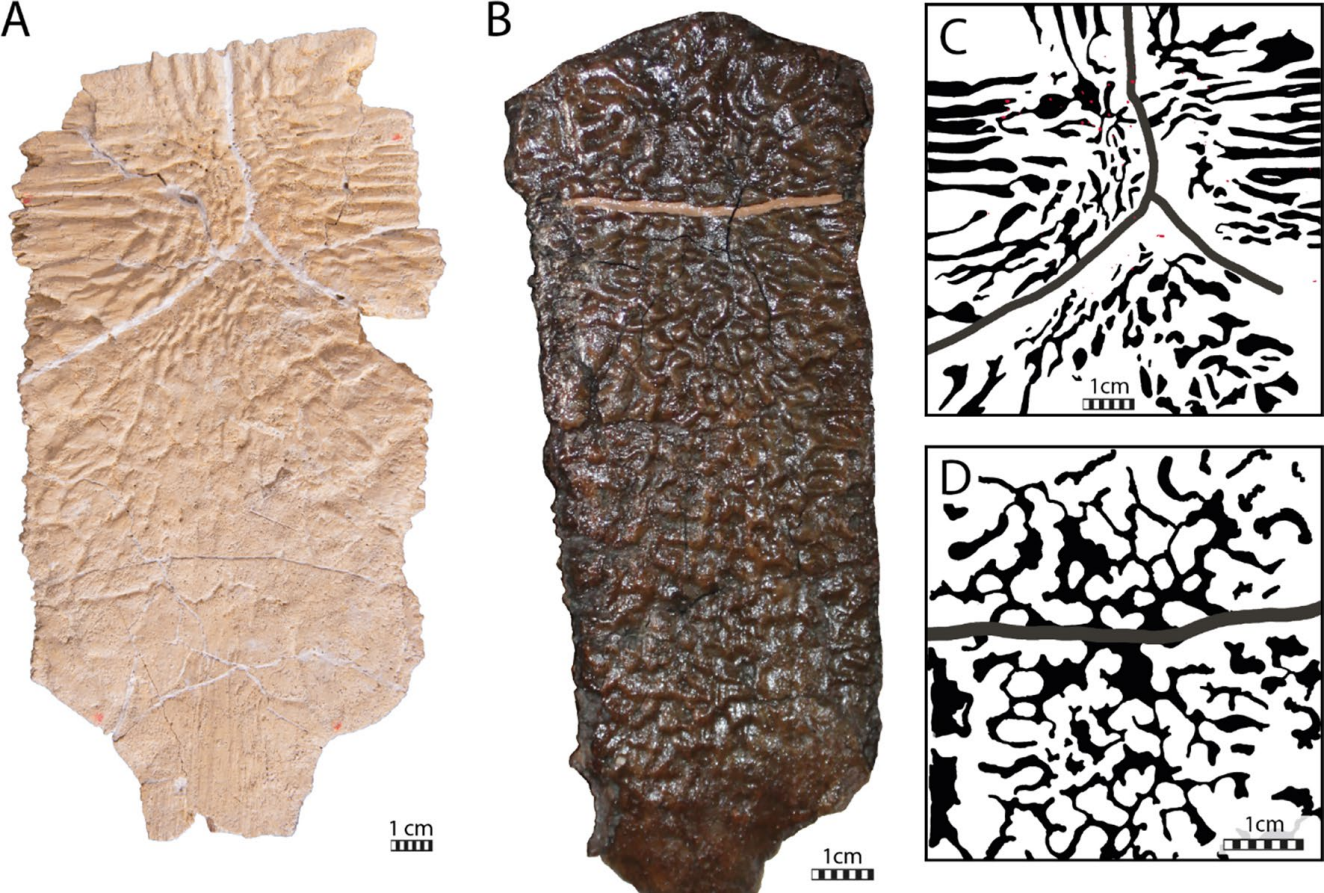
Comparisons of texturing patterns of *Glyptochelone suyckerbuykii* and *Peritresius ornatus*: **A** third right costal of *Glyptochelone suyckerbuykii* IRSNB R507. **B** Third right costal of *Peritresius ornatus* NJSM 11051. **C** Interpretative drawing of the texturing pattern of *Glyptochelone suyckerbuykii* IRSNB R507. **D** Interpretative drawing of the texturing pattern of *Peritresius ornatus* NJSM 11051.The negative relief is figured in black, the positive relief is figured in white, the scale sulci are figured in grey, and the nutritive foramina are figured in red

The best-known ornamented Late Cretaceous sea turtle is undoubtedly *Peritresius ornatus* (Leidy, 1856; Fig. 15), a ctenochelyid which occurs in Maastrichtian Redbank (Hay, 1908) and Navesink formations of New Jersey (Baird, 1964). Some fragmentary material from the Maastrichtian Prairie Bluff Chalk of Mississippi and from the Late Campanian Ripley Formation, Demopolis Chalk, and Mooreville Chalk of Alabama and Mississippi with a similar texture pattern were reported more recently (Gentry et al., 2018), but the attribution to *Peritresius ornatus* is somewhat tenuous given their fragmentary nature (see Gentry et al., 2018, Figs. 7, 8). Despite the superficial similarities between the ornamentations found in *Peritresius ornatus* and *Glyptochelone suyckerbuykii*, several differences are observable, potentially allowing for the diagnosis of fragmentary remains. First, the positive relief in *Peritresius ornatus* is only formed by rounded vermiculations. The sharp crests found in *Glyptochelone suyckerbuykii* are absent. Second, the spherical foramina that interrupt the groves around the ossification centers of the bony plates of *Glyptochelone suyckerbuykii* are absent in *Peritresius ornatus*. Third, in *Glyptochelone suyckerbuykii* the length and shape of crests is much more heterogeneous within a given bone plate, with a tendency to elongate and straiten drastically away from the ossification center and prolongate to the adjacent plates (Figs. 2A–C, 7E, 9A–B, 15A, C). This does not occur in *Peritresius ornatus* (see Fig.  1 C in Baird, 1964; Fig. 15B, D). And fourth, in *Glyptochelone suyckerbuykii* the pattern radiates much more clearly from the ossification center than in *Peritresius ornatus*.

Outside of the Atlantic realm, ornamented fragments of chelonioid sea turtles have been reported from Late Cretaceous deposits of Kazakhstan (Averianov, 2002) and Russia (Averianov & Yarkov, 2004; Danilov, 2018). Among these, an isolated anterior neural from the Campanian Eginsai Formation of Kazakhstan, ZISP PH 8/37, was initially figured and tentatively attributed to *Turgaiscapha kushmurunica* (Averianov, 2002) but later identified as *Cheloniidae* indet. (Danilov, 2018). This fragment displays an ornamentation similar to *Glyptochelone suyckerbuykii* consisting in radiating crests, vermiculation, and deep rounded foramina around the center of the bone (compare Averianov, 2002 and Fig. 9).

Even though the neural arch is not preserved, the hexagonal shape and dorsal face of ZISP PH 8/37 is similar to the neurals 2–4 of IRSNB R507, elements not associated with interneurals. It is therefore likely that ZISP PH 8/37 belongs to *Glyptochelone suyckerbuykii* as conceived herein, even though its fragmentary nature prevents us to be certain of this attribution.

Ornamented bones also occur in some coeval marine pleurodire turtles, such as the bothremydine *Abalakemys chapmanae* from the upper Maastrichtian of Niger (Pérez-García, 2023) and the taphrosphine *Taphrosphys sulcatus* from the lower Danian Hornerstown Formation of New Jersey (Gaffney et al., 2006). The ornamentation of these taxa, however, can easily be differentiated from that of *Glyptochelone suyckerbuykii* in that *Abalakemys chapmanae* displays short and low vermiculations (Pérez-García et al., 2023) and *Taphrosphys sulcatus* is carved by a low network of interlacing fine grooves or furrows that are separated by flat polygons instead of the acute crest found in *Glyptochelone suyckerbuykii*.

An intriguing Paleogene taxon that exhibits deep ornamentations that are restricted to the vertebral scales is *Osonachelus decorata* (Lapparent de Broin et al., 2014, 2018) from the Bartonian of Spain. The surface texture of this taxon consists of thick vermiculations and wide pitting that contrasts the pattern found in *Glyptochelone suyckerbuykii* by its random subdivisions within the vertebral scale (compare Fig. 2C with Lapparent de Broin et al., 2014, Fig. 8C). Some Neogene cheloniids, such as *Trachyaspis lardyi* (Meyer, 1843), in particular some specimens from the Middle Miocene of Japan (Hasegawa, 2005), also display also a deep ornamental pattern that is reminiscent of *Glyptochelone suyckerbuykii*, by consisting of an alternation of crests and grooves that radiate from the ossification center of the bones and become straighter and longer towards the edges of the bones (compare Fig. 2A, C with Hasegawa, 2005), but given the difference in stratigraphic age, this is likely a convergence.

### Neural homology in turtles

**Fig. 16:**
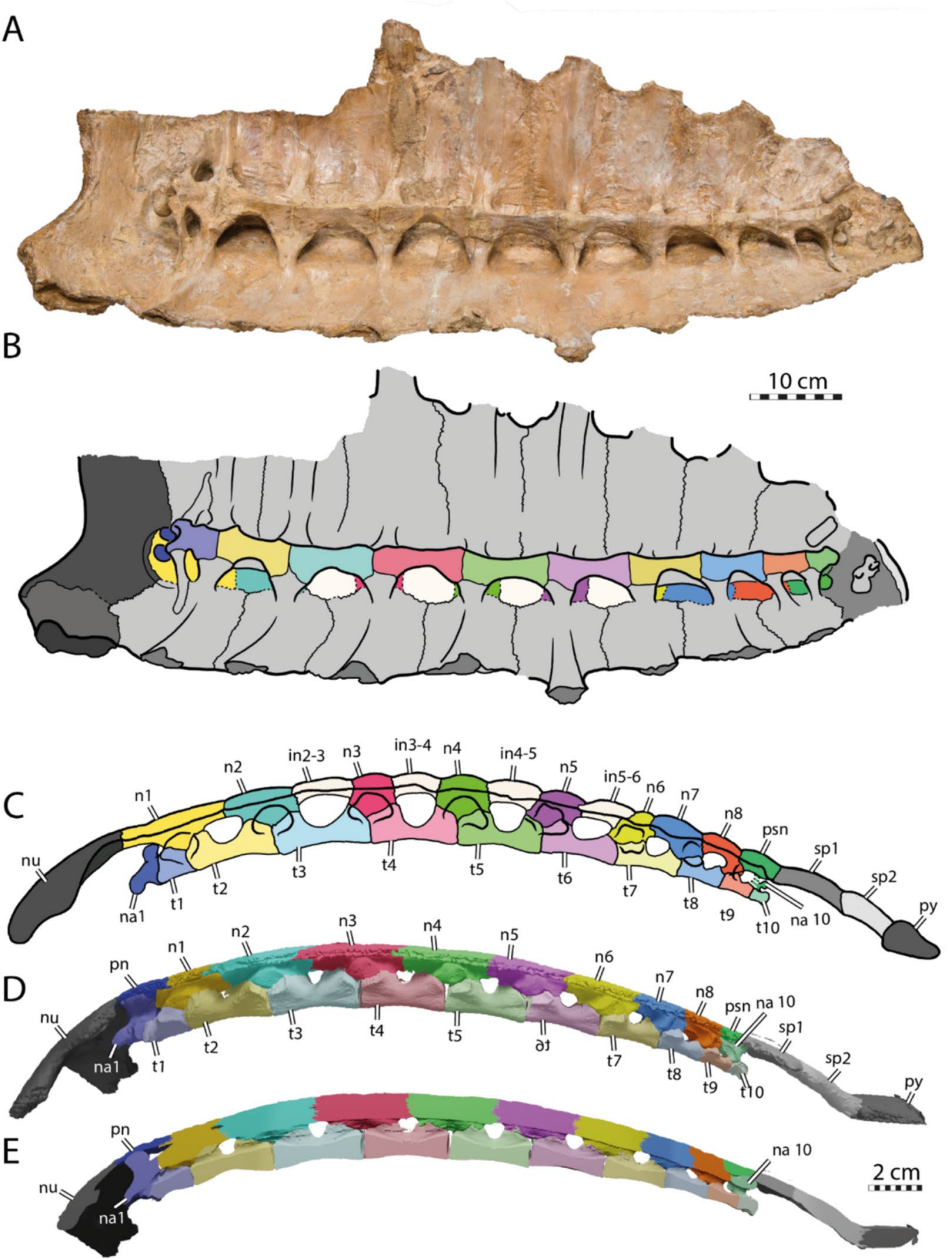
Comparison of neural series between *Glyptochelone suyckerbuykii* IRSNB R2 and *Chelonia mydas* SMF63250. **A** Ventral view and **B** interpretive line drawing of the thoracic vertebrae of *Glyptochelone suyckerbuykii* IRSNB R2. **C** Reconstruction of the contacts in lateral view of the thoracic vertebrae of *Glyptochelone suyckerbuykii* IRSNB R2. The suprapygal and pygal are based on NHMM 4548. **D** Lateral view and **E** sagittal section of the thoracic vertebrae of *Chelonia mydas* SMF 63250. *in* interneural, *na* neural arch, *n* neural, *nu* nuchal, *pn* preneural, *psn* postneural, *py* pygal, *sp* suprapygal, *t* thoracic vertebral centrum

Some carapacial elements of the midline column (i.e., preneurals, neurals, interneurals, postneurals, and suprapygals) show much variation across turtles, which has been used for decades to infer phylogenetic relationships (e.g., Evers & Benson, 2019; Gaffney et al., 2006; Hirayama, 1985, 1998; Meylan, 1987). However, uncertainty exists regarding their homology, especially the nature of the preneurals, interneurals, and postneurals, and the distinction between neurals and suprapygals (Anquetin et al., 2014; Cherepanov, 2016; Pritchard, 1988; Zangerl, 1969). We here explore a possible new criterion for establishing the homology of neural elements and phrase conjectures as to the primary causes of variation. A central pillar herein is the relatively novel realization that the neurals are not independent ossifications but rather represent hypertrophied neural arches that contribute to the surface of the shell (e.g., Cherepanov, 1989; Cherepanov & Danilov, 2024; Hirasawa et al., 2013; Scheyer et al., 2008; Fig. 16). As the number of thoracic vertebrae does not vary among turtles (Müller et al., 2010), the relationship with the neural arches can thus be used to establish homologies.

**Fig. 17:**
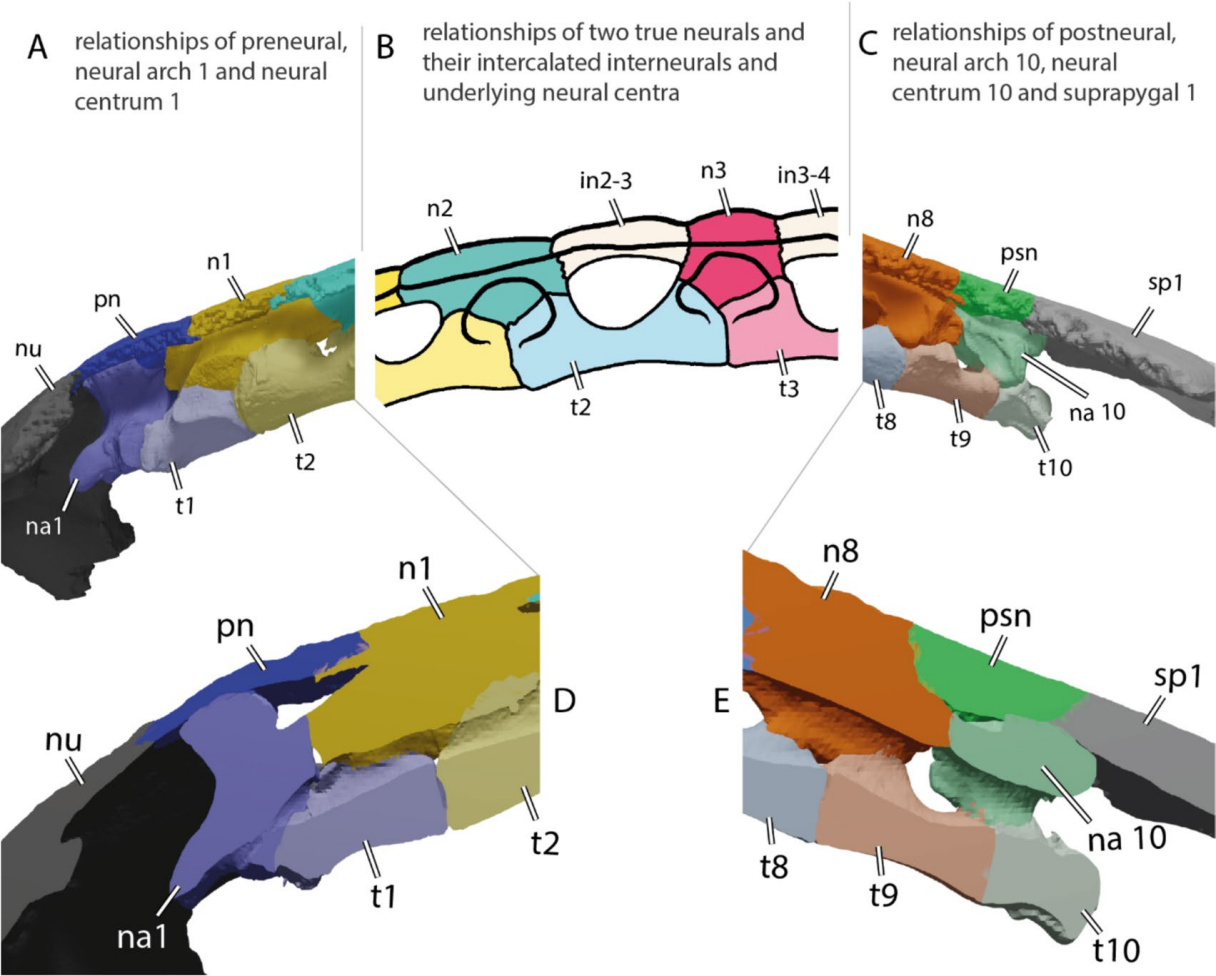
Close-ups from Fig. 16 that illustrate the relationships between the preneural, interneurals, and the postneural with the surrounding bones. **A**, **C** 3D models and **D**, **E** sagittal sections of *Chelonia mydas* SMF 63250. **B** reconstruction of *Glyptochelone suyckerbuykii* IRSNB R2. *in* interneural, *na* neural arch, *n* neural, *nu* nuchal, *pn* preneural, *psn* postneural, *sp* suprapygal, *t* thoracic vertebral centrum

**Fig. 18:**
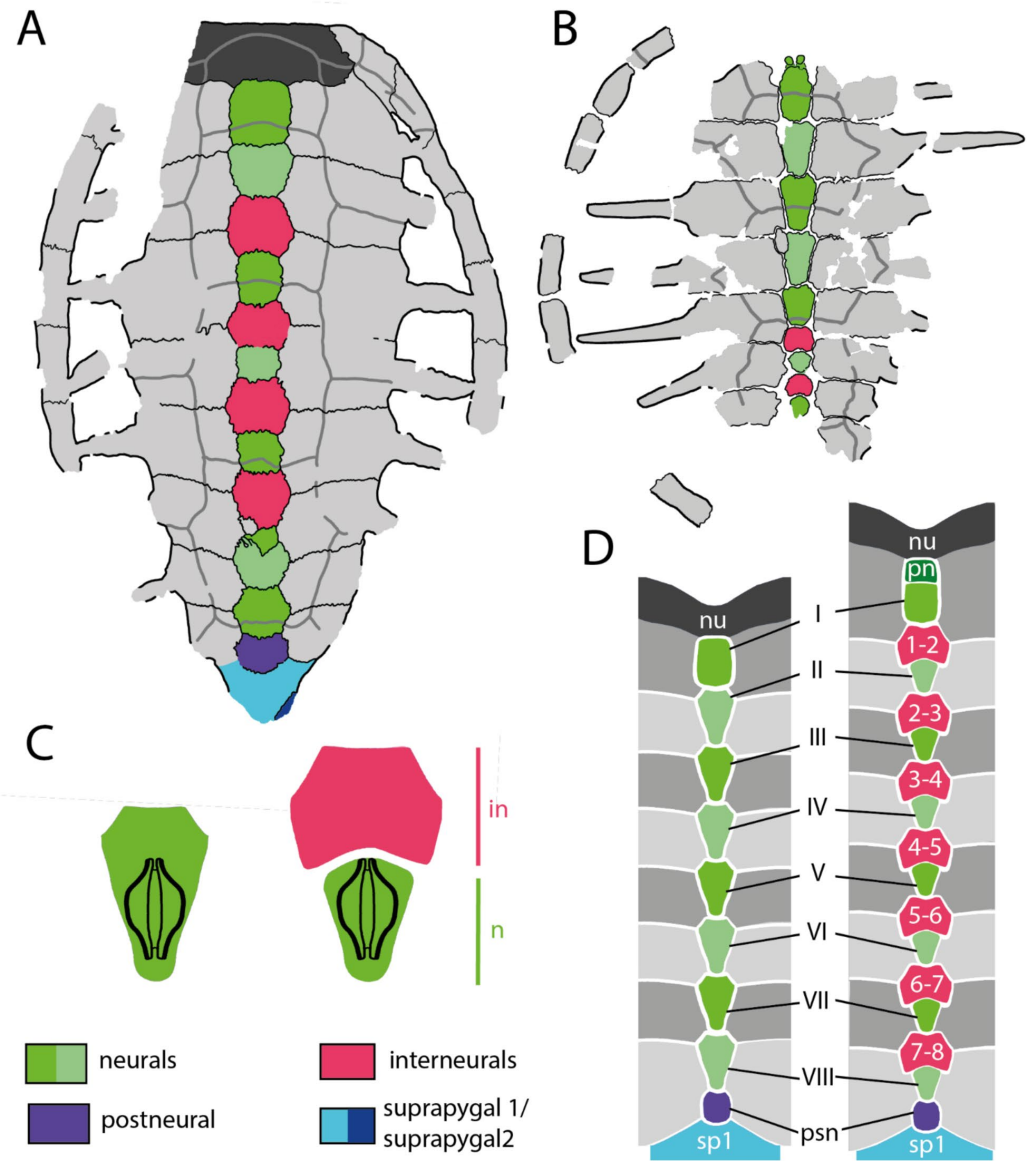
Homologies of neurals and interneurals. **A** Interpretative drawing of the neural series of IRSNB R2. **B** Interpretative drawing of the neural series of IRSNB R507. **C** schematic ventral views of “regular neural” and “neural + interneural” condition. **D** Schematic dorsal views of neural series with absence and presence of interneurals and assessed homology relationships

In this study we note that the neural series of *Glyptochelone suyckerbuykii* consists of an alternance of larger, free elements (i.e., the interneurals) and smaller plates that are expansions of the neural arches (i.e., the neurals; Figs. 2, 9, 10, 16, 17, 18A, B). A similar arrangement was revealed by Zangerl and Turnbull (1955) for the Miocene *Procolpochelys grandaeva* in which the regular, elongated, hexagonal neurals (6A shape of Pritchard, 1988) of other chelonioids appear to be replaced by a large anterior interneural and a small, intermediate posterior neural, the same pattern is also present in modern cheloniids (e.g., *Lepidochelys olivacea*, see Additional file 8, Fig. 2). Nonetheless, there seems to be a generality that interneurals tends to be larger and neurals smaller. In contrast to Zangerl and Turnbull (1955), we suggest reserving the name neural for the neural arch outgrowth, as its genesis is homologous to the neurals of other turtles. For the intermediate element, by contrast, we suggest the name interneural.

We are unaware of any literature documenting the formation of a turtle shell through the subdivision of already formed elements as was hypothesized by Zangerl and Turnbull (1955) for *Procolpochelys grandaeva*. Instead, studies seem to agree that all elements, including supernumerary ones, originate early in ontogeny and then expand in size across the surface of the shell to form a more or less continuous structure (e.g., Cherepanov, 2016; Gilbert et al., 2001; Sheil & Greenbaum, 2005; Werneburg et al., 2009). So, while the possibility exists that interneural elements split from an adjacent neurals, we find it more plausible to postulate that they represent neomorphic elements that are not homologous with true neurals. Although not fully equivalent, this is broadly consistent with observation of Cherepanov (2016) that neomorphic “intercalary elements” form in fontanelles during development. Interestingly, such supernumerary ossifications commonly precipitate into the neurocostal fontanelles of extant tortoises (Cherepanov, 2016) and both IRSNB R2 and R507 display such elements that disturb their midline symmetry. Following this hypothesis, we suggest numbering interneural elements by reference to both neighboring neurals, because there is no evidence that the interneural splits from a particular neural. We note that all turtles with interneurals display relatively elongate shells. It is therefore possible that there is a relationship between the shape of the shell and the formation of interneurals. We encourage testing our assertions by studying the late embryology and early postnatal ontogeny of turtles with common interneurals, such as *Lepidochelys* spp.

Epineurals, yet another type of supernumerary midline element, have previously been reported for the shells of fossil pan-chelonioids, in particular ctenochelyids (e.g., Gentry, 2018). Epineurals are laterally compressed, conical bones, found along the midline of taxa with pronounced carapacial keels. They are universally situated on top of the dorsal aspect of two consecutive neurals (e.g., Case, 1898; Gentry, 2018; Hay, 1898; Wieland, 1905). Given the topological position of these elements above regular neurals, most recent authors agree on characterizing their position as “epithecal” (Cherepanov & Danilov, 2024; Gentry, 2018), but phylogenies, such as ours, place doubt on a possible homology with the “epithecal” carapace of dermochelyids. Although epineurals and interneurals occur within the neural series between two successive neurals, we note two significant differences between these two categories of plates. First, epineurals exclusively occur at the posterior border of vertebral scutes (e.g., Baird, 1964; Gentry, 2018; Hay, 1908; Wieland, 1905) whereas interneurals freely occur throughout the full neural series (Figs. 2, 9, 16, 17); Deraniyagala, 1939; Zangerl & Turnbull, 1955). Second, epineurals are distinct, conical elements that lack visceral surfaces and are situated above the neurals while interneurals are flat elements, with broad visceral surfaces, and flush with the surrounding carapacial bones. As no extant turtle possesses epineurals, it will never be possible to ascertain with confidence how these elements formed, even if their proximity with the posterior edge of vertebral scales suggest that they are related with the presence of high keeled vertebrate scutes. Nonetheless, even if the genesis of epineurals and interneurals may be developmentally homologous, our phylogeny suggests that they are not phylogenetically homologous (i.e., synapomorphic).

A particular type of variation to the neural series is the occurrence of a preneural. Across turtles, this element has consistently or variably been reported in Triassic stem turtles (Szczygielski & Sulej, 2019), baenids (e.g., Gilmore, 1919; Hay, 1906), meiolaniforms (e.g., Sterli et al., 2015), thalassochelydians (Püntener et al., 2017), sinemydids (e.g., Brinkman & Peng, 1993; Tong et al., 2004), carettochelyids (e.g., Joyce, 2014), pan-trionychids (e.g., Carpenter, 1981; Cherepanov, 1995; Meylan, 1987), and, most notable for this contribution, stem-chelonioids (e.g., Gentry, 2017; Zangerl, 1953a) and crown cheloniids (e.g., Zangerl, 1969). Incidentally, it is not fully clear if all listed taxa exhibit true preneurals, as the first neural of fossil turtles tend to break where it is crossed by the vertebral I/II sulcus, but reevaluating all reports is outside the scope of the present study.

Previous workers investigated the nature of preneurals in pan-trionychids, with some authors considering this element to be homologous with the first neural of other turtles (e.g., Meylan, 1987), others suggesting to be a “true” first neural as it is associated with the first thoracic vertebra (e.g., Kordikova, 2000, 2002), and yet others concluding it to be a neomorphic bone (e.g., Cherepanov, 1995). Joyce (2025a) more recently summarized the available literature and sided with Cherepanov (1995) that the preneural represents a neomorph, as it is associated with the first thoracic vertebra, but is not an outgrowth of the first thoracic neural arch. Instead, the association is via a sutural contact. This resembles the condition we here observe for an extant cheloniid (*Chelonia mydas* SMF 63250; Figs. 16D, E, 17; Additional file 9). As such, the preneural of pan-trionychids and cheloniids resembles the interneurals of marine turtles by being a supernumerary ossification but differs by being located above the first neural arch and, in the case of cheloniids, by displaying a sutured contact with the first neural arch. Though the argument could be made that the preneural is yet another interneural, we retain the term because of its historical precedence and because of its relationship with the first neural arch (Fig. 17).

The number of posterior neural and suprapygal elements is commonly used as a phylogenetic character, but little attention has been accorded to clarifying the homology of supernumerary elements. Mesozoic helochelydrids have a tendency to exhibit additional elements in the pygal region, which have so far been interpreted as a third suprapygal (e.g., Joyce et al., 2014; Joyce, 2025b; Pérez-García et al., 2020). Riggs (1906) reported a Late Cretaceous nanhsiungchelyid with a small, supernumerary element, which he labelled as a postneural. Among thalassochelydians, many plesiochelyids exhibit an additional bone termed the “intermediate element” by Anquetin et al. (2014) and Püntener et al. (2017). Finally, many extant chelonioids are reported to display additional neurals or suprapygals (e.g., Deraniyagala, 1939; Zangerl & Turnbull, 1955). Pritchard (1988) reported that the posterior supernumerary element of *Chelonia mydas* is sutured to the neural arch of the tenth thoracic vertebra, an observation we are able to reproduce herein based on a different specimen (Figs. 16D–E, 17; Additional file 9). As the suprapygals are differentiated from neurals by not being expansions of the neural arches, this suggests that the supernumerary element of *Chelonia mydas* is neither a true neural, nor a true suprapygal, and thus somewhat equivalent in its development to the preneural by being sutured to a neural arch (Fig. 17). Although the term “postneural” was used by some in the late 19th and early 20th for suprapygals (e.g., Baur, 1889; Reinach, 1900), we suggest using this term for supernumerary element sutured with the tenth thoracic vertebra, to reflect similarities with the preneural.

Although the relevant region is covered by matrix in *Glyptochelone suyckerbuykii* IRSN R2 (Figs. 2A, C, 16A–C), the possibility cannot be excluded that the supernumerary element in this taxon is a true ninth neural, even if a true ninth neural has not yet been documented for any Late Cretaceous pan-chelonioid. We urge the reinterpretation of supernumerary elements in other taxa, particular thalassochelydians, while speculating that these most likely represent postneurals as well.

In contrast to the above discussed formation of supernumerary elements, many turtles display a loss of neurals. The limited amount of literature pertaining to this subject suggests that the neural arches of some taxa simply do not rise to contribute to the surface of the carapace (Pritchard, 1988; Scheyer et al., 2008; Smales, 2019; Thomson and George, 1996). A peculiar reduction was documented by Cherepanov (1989) for a specimen of the extant testudinid *Testudo graeca*, where the seventh neural arch is the last to rise to the surface to form a true neural (i.e., neural VI), but the remaining space is filled with four elements, of which the anterior two roof the arches of neurals VII–X and therefore partially fulfil our criteria of postneurals. It is not clear if this observation is broadly representative for testudinids, or even testudinoids, but, if so, it suggests that these animals have an expanded set of postneural and suprapygal elements at the cost of true neurals, which in return might explain particularly high levels of variability and unusual neural connections observed in the pygal region of these animals (e.g., Cherepanov, 2016; Garbin et al., 2018; Joyce & Bell, 2004; Vlachos & Rabi, 2018). We suggest further investigation of this character complex, specifically for testudinoids.

In conclusion, we suggest reclassification of midline elements to the shell of turtles using the following scheme:

Preneural: A midline plate situated posterior to the nuchal and anterior to the neural series that caps or suturally connects to the neural arch of the first thoracic vertebra.

Neural: The dorsal outgrowth of a neural arch that contributes to the external surface of the shell along the midline. As neurals represent neural arch expansions, they lack a suture with their corresponding neural arches. The numbering of neurals, by tradition, is offset from the neural arch to which is corresponds (e.g., neural arch II = neural I; see Kordikova, 2002, for an alternative counting system where the numbering of neurals and neural arches is coordinated).

Interneural: A midline plate that is situated between two neurals and does not represent a neural arch expansion. Interneurals thus at best might have sutural connections with neural arches. Interneurals are numbered by reference to the two surrounding neurals. When present, interneurals are located at the junction of two pairs of costals and are typically larger than the neurals.

Postneural: A midline plate situated posteriorly to the neural series that caps the tenth neural arch and displays a sutured contact with it.

Supraygal: An independent midline plate not associated with the dorsal vertebral column, lacking any contact with the neural arches and generally displaying a wider shape than the other elements of the neural series.

The strict application of the nomenclature proposed above demands examination of the visceral aspects of shells, but such data is not available for many taxa, in particular fossils. However, we note that neurals can typically be differentiated from interneurals by their position relative to the costals. In particular, as genuine neurals are outgrowths of the neural arches, which articulate with the costal ribs, it is the neurals that align with the costals whenever interneurals are present and the interneurals are placed at the junctions of two pairs of costals (Fig. 18). We are not able to develop similar rules of thumb for the identification of pre- and postneurals.

We introduced three new characters that implement our observations in regard to the presence and development of interneurals, preneurals, and postneurals (see Materials and methods). Our phylogenetic analysis performed with implied weighting (k = 12) demonstrates that this introduction of new characters to the matrix did not substantially affect the topology. Indeed, the only change displayed by the tree obtained with the new characters disabled affects the dermochelyid group in which *Glyptochelone suyckerbuykii*, *Erquelinnesia gosseleti*, *Procolpochelys charlestonensis* and *Oligochelone rupeliensis* form an unresolved polytomy instead of placing *Glyptochelone suyckerbuykii* and *Procolpochelys charlestonensis* as sister taxa and *Erquelinnesia gosseleti* at the basis of the latter clade (Additional file 8, Fig. 7). Although, the addition of the new characters resulted in a better resolved tree (Additional file 8, Fig. 7), the retrieval of *Glyptochelone suyckerbuykii* and *Procolpochelys charlestonensis* as sister taxa based only on interneural characters (see above) is questionable as the available skull material of *Procolpochelys charlestonensis* greatly resembles that of extant cheloniids (Weems & Brown, 2017) which polymorphically develop interneurals as well. So, while our revised homology may be helpful in aiding communication and further conceptualizing the development of shell variation, it does not appear to be useful for the moment in further resolving pan-cheloniid relationships.

## Supplementary Information

Additional file 1. Updated list of characters.

Additional file 2. Updated matrix.

Additional file 3. All of the MPTs obtained without implied weighting.

Additional file 4. All of the MPTs obtained with implied weighting k=12.

Additional file 5. All of the MPTs obtained with implied weighting k=7.

Additional file 6. List of synapomorphies for k=12.

Additional file 7. List of synapomorphies for k=7.

Additional file 8. Supplementary figures including additional pictures of *Glyptochelone suyckerbuykii *IRSNB R2, *Allopleuron hofmanni* specimens, and previously unfigured extant cheloniid material.

Additional file 9. Description of neural series of Chelonia mydas.

## Data Availability

Supplementary Information The online version contains supplementary material available at xxx Additional file 1. Updated list of characters Additional file 2. Updated matrix Additional file 3. All of the MPTs obtained without implied weighting Additional file 4. All of the MPTs obtained with implied weighting k = 12 Additional file 5. All of the MPTs obtained with implied weighting k = 7 Additional file 6. List of synapomorphies for k = 12 Additional file 7. List of synapomorphies for k = 7. Additional file 8. Supplementary figures including additional pictures of Glyptochelone suyckerbuykii IRSNB R2, Allopleuron hofmanni specimens, and previously unfigured extant cheloniid material. Additional file 9. Description of neural series of Chelonia mydas (SMF 63250).

## Abbreviations

AMNH: American Museum of Natural History, New York, New York, USA
FMNH: Field Museum of Natural History, Chicago, Illinois, USA
IRSNB: Institut Royal des Sciences Naturelles de Belgique, Brussels, Belgium
NHMM: Natuurhistorisch Museum Maastricht, Maastricht, Netherlands
NJSM: New Jersey State Museum, Trenton, New Jersey, USA
NTUM: National Taïwan University Museum Group, Taipei, Taïwan
QM: Queensland Museum, Brisbane, Queensland, Australia
SMF: Senckenberg Museum Frankfurt, Frankfurt, Germany
UF: Florida Museum of Natural History, Gainesville, Florida, USA
USNM: Smithsonian National Museum of Natural History, Washington D.C., USA
YPM: Yale Peabody Museum, New Haven, Connecticut, USA
ZISP: Zoological Institute of the Russian Academy of Sciences, Saint Petersburg, Russia
