# Supplementary material for: A redescription of Glyptochelone suyckerbuykii (Ubaghs, 1879), an enigmatic fossil sea turtle (Chelonioidea) from the Maastrichtian of the Netherlands and Belgium, sheds new light on fossil sea turtle shell variation and neural bone homology
Source: Swiss J Palaeontol. 2025 Oct 1;144(1):62. doi: 10.1186/s13358-025-00389-y (PMC12488835; doi:10.1186/s13358-025-00389-y)
Supplement: Supplementary file 8 — Additional file 8. Supplementary figures including additional pictures of Glyptochelone suyckerbuykii IRSNB R2, Allopleuron hofmanni specimens, and previously unfigured extant cheloniid material. [file 13358_2025_389_MOESM8_ESM.docx]

**Supplementary figures**


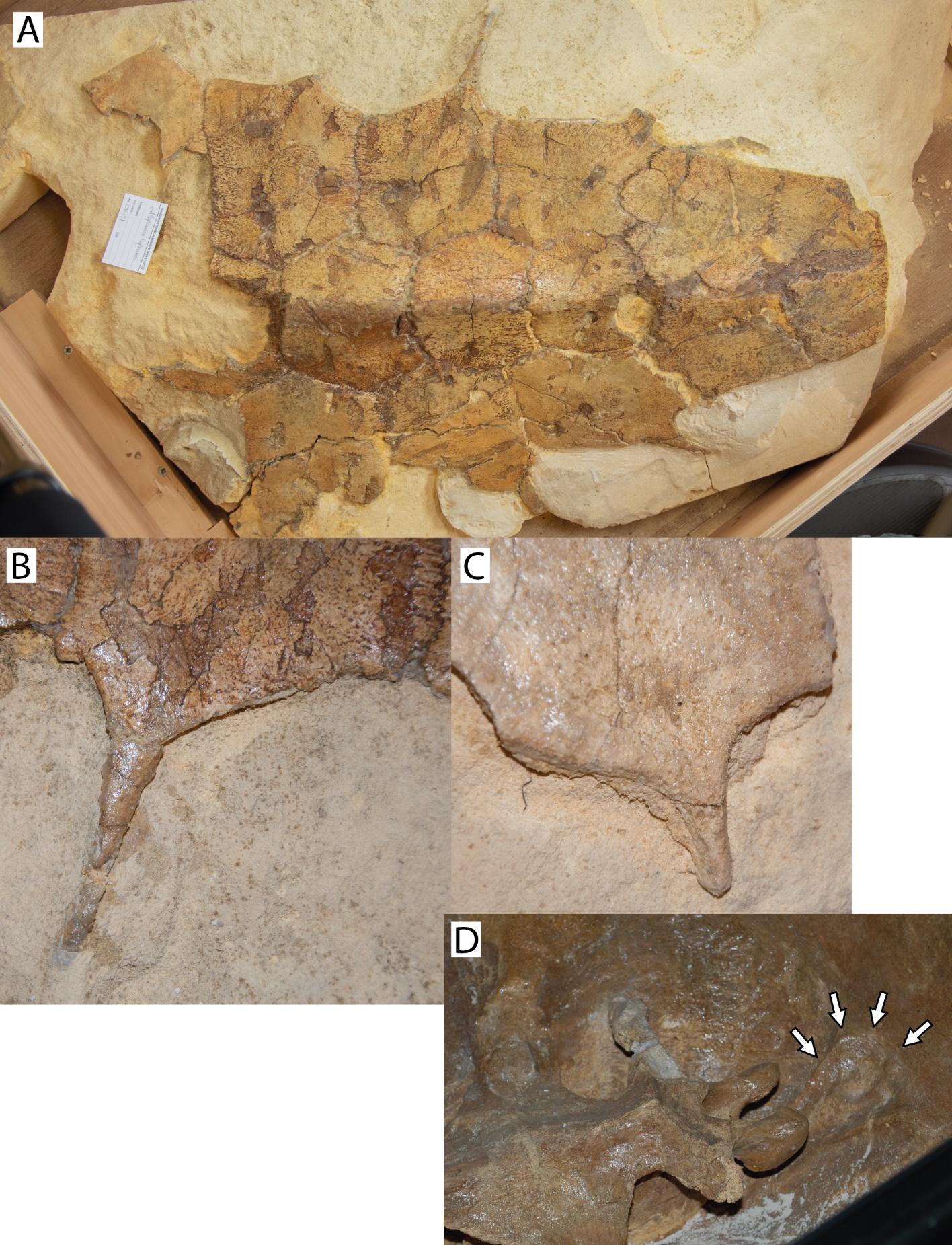
**Figure.1** Additional pictures of *Allopleuron hofmanni*: NHMM 2010157 A. Dorsal view; B. and C., Close up on a costals; D. NHMM 9016, close up on the visceral face of the nuchal showing the depression.


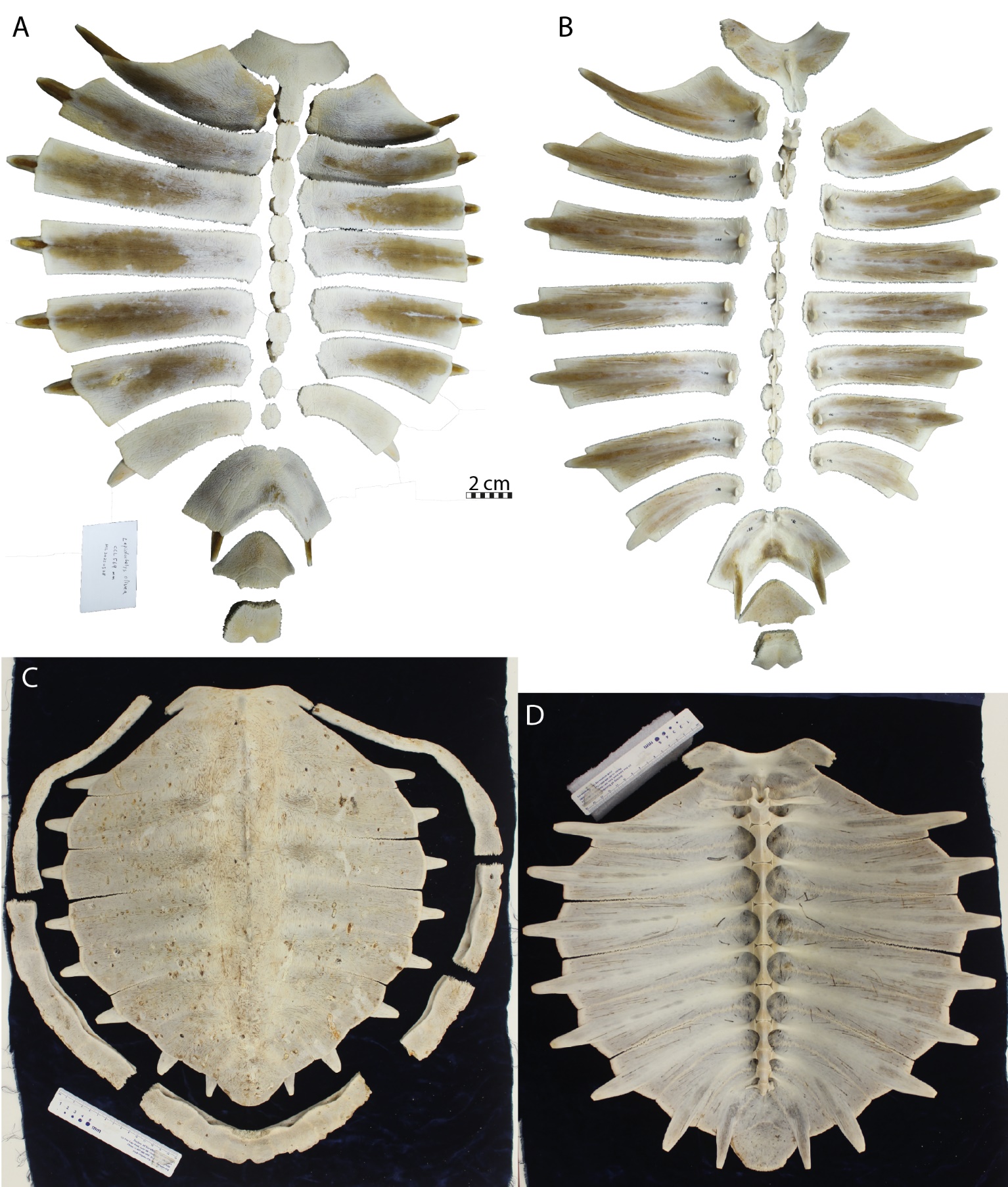


**Figure.2** Photography of *Lepidochelys olivacea* shells showing presence of interneural within the neural series. A. CCL 569 dorsal view; B. CCL 569 visceral view; C. QM J85545 dorsal view; D. QM J85545 visceral view.


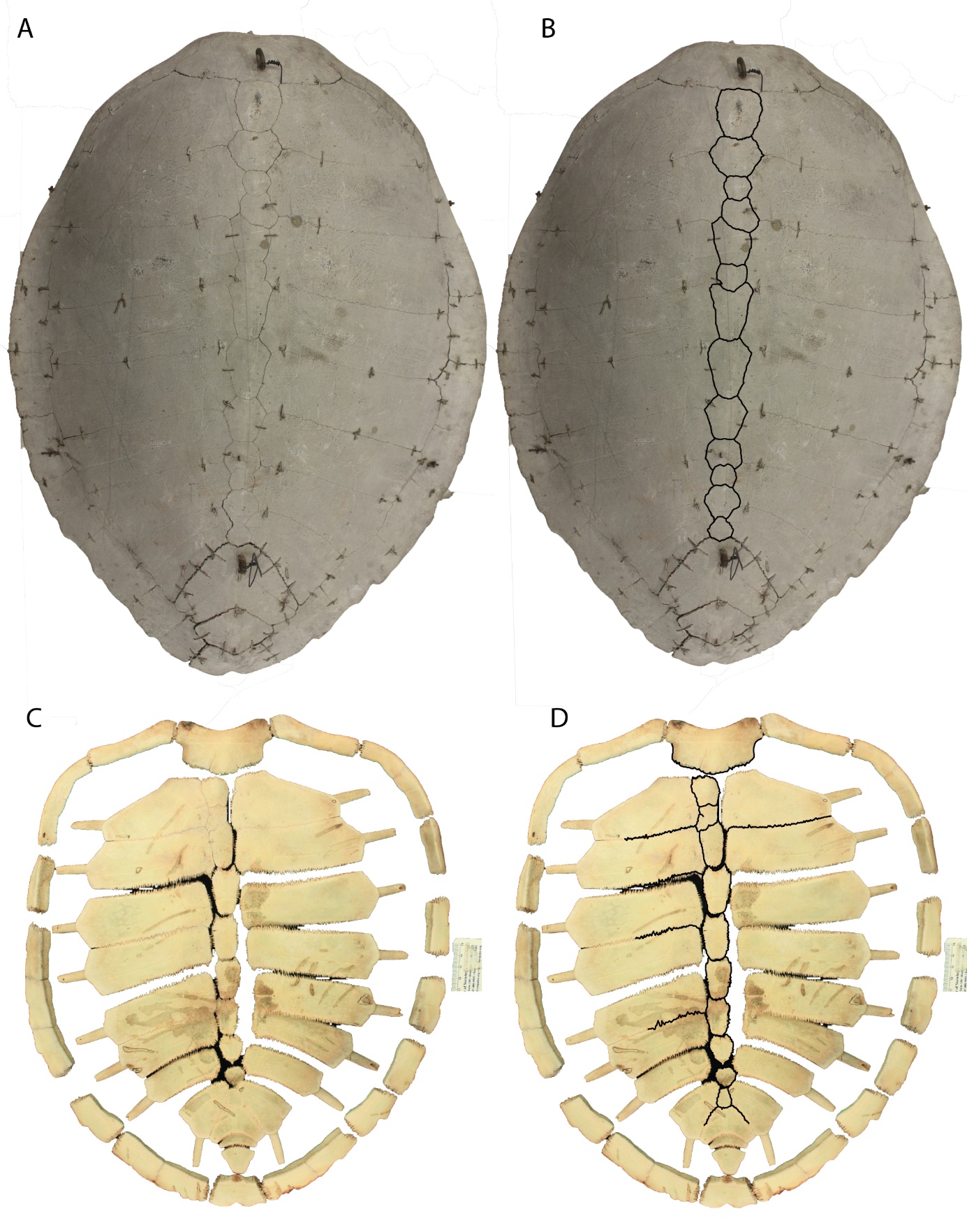
**Figure.3** Additional pictures of *Natator depressus* showing the variation of the number of elements in neural series A. QM J14463 dorsal view; B. interpretative schema of the neural series QM J14463; C., QM J92457 dorsal view; D. interpretative schema of the neural series QM J92457.


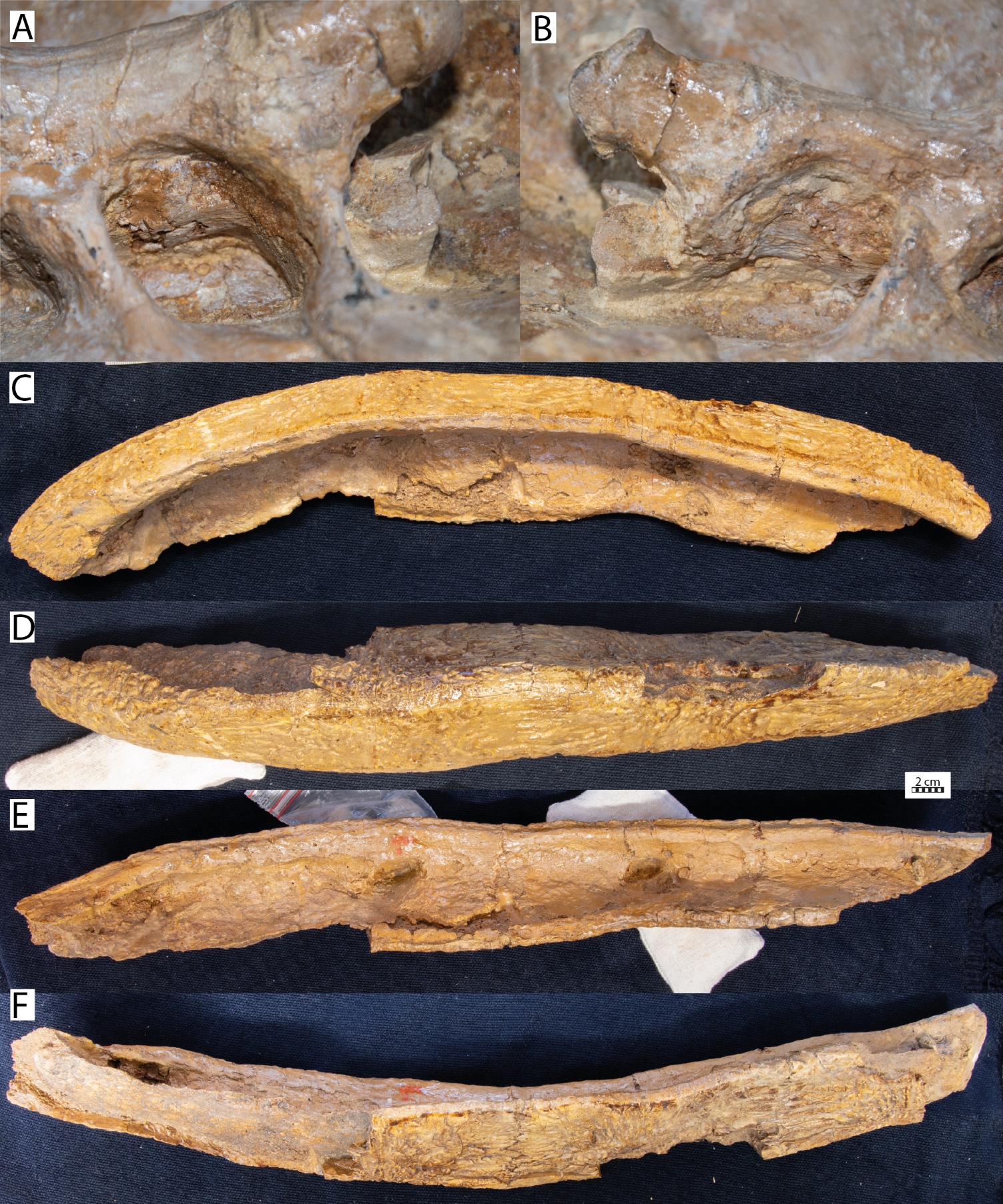


**Figure.4** Additional pictures of *Glyptochelone suyckerbuykii* IRSNB R2. A. Zoom on the tenth thoracic vertebrae lateral right view; B. Zoom on the tenth thoracic vertebrae lateral left view. Left peripherals 3 to 6: C. ventral view; D. Lateral view; E. medial view; F. dorsal view.


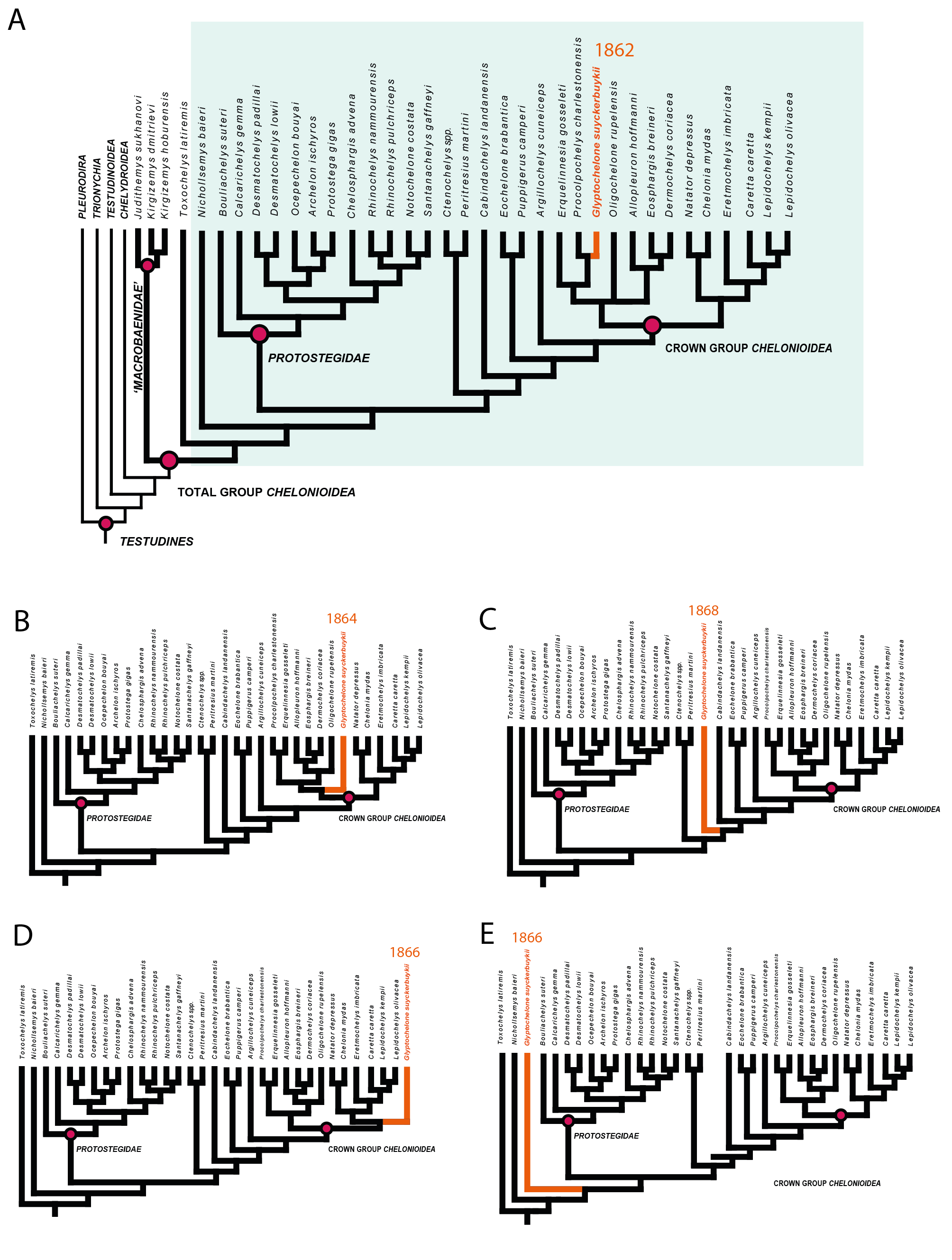
­**Figure.5** Variations of the tree length in function of the diverses positions of *Glyptochelone suyckerbuykii*. A. Portion of the tree 3 used for character optimization and tree length obtained on mesquite; B. placed at the stem of dermochelyids; C. result for *Glyptochelone suyckerbuykii* placed one stem more crownward than ctenochelyids; D. result for *Glyptochelone suyckerbuykii* placed at the stem of cheloniids; E. result for *Glyptochelone suyckerbuykii* placed one step more stemward than protostegids.


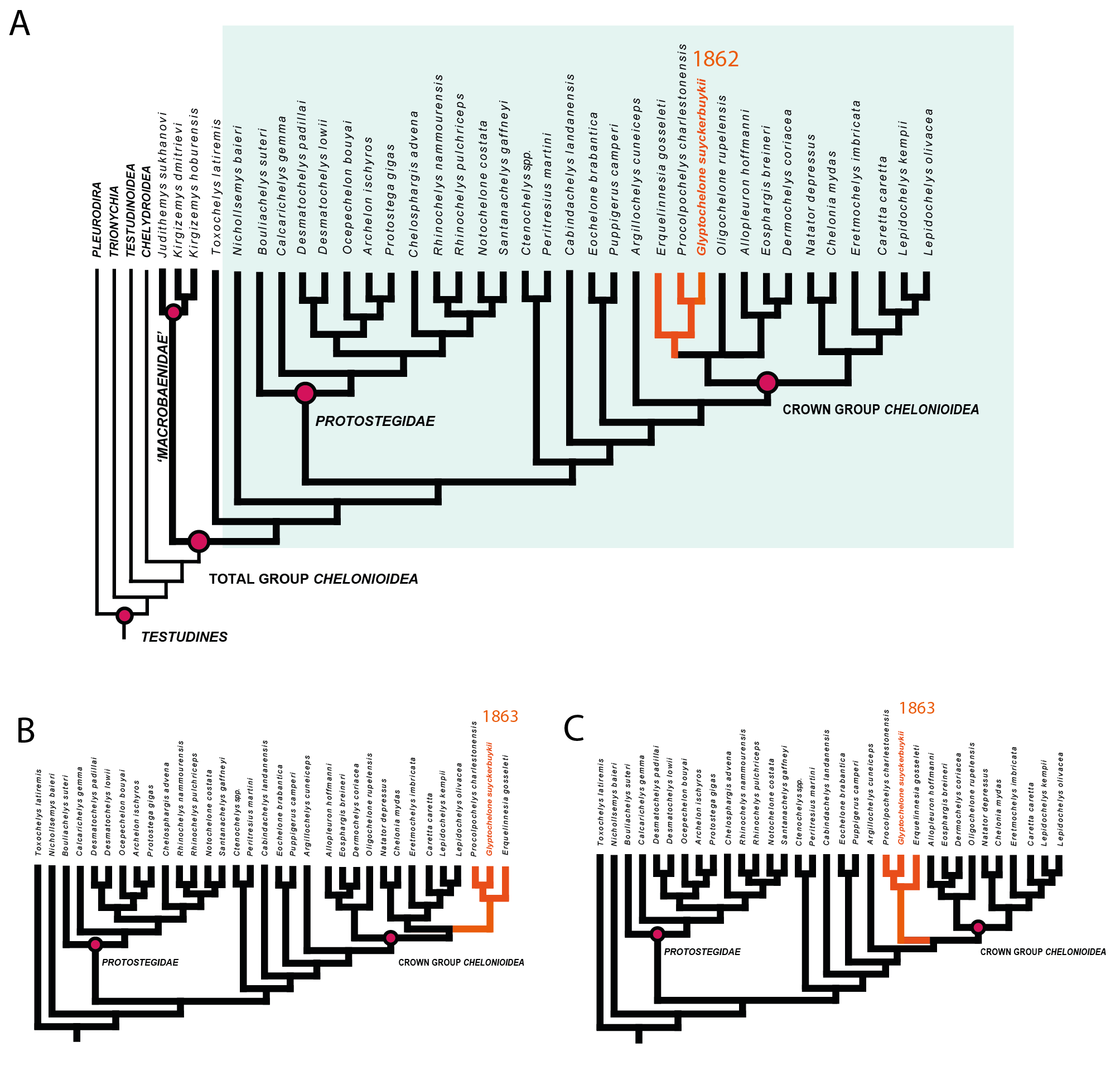


**Figure.6** Variations of the tree length in function of the diverses positions of the group (*Glyptochelone suyckerbuykii* + *Erquelinnesia gosseleti* + *Procolpochelys charlestonensis*). A. Portion of the tree 3 used for character optimization and tree length obtained on mesquite; B. result for (*Glyptochelone suyckerbuykii* + *Erquelinnesia gosseleti* + *Procolpochelys charlestonensis*) placed at the stem of cheloniids; C. result for (*Glyptochelone suyckerbuykii* + *Erquelinnesia gosseleti* + *Procolpochelys charlestonensis*) placed at the stem of crown-chelonioids.

**
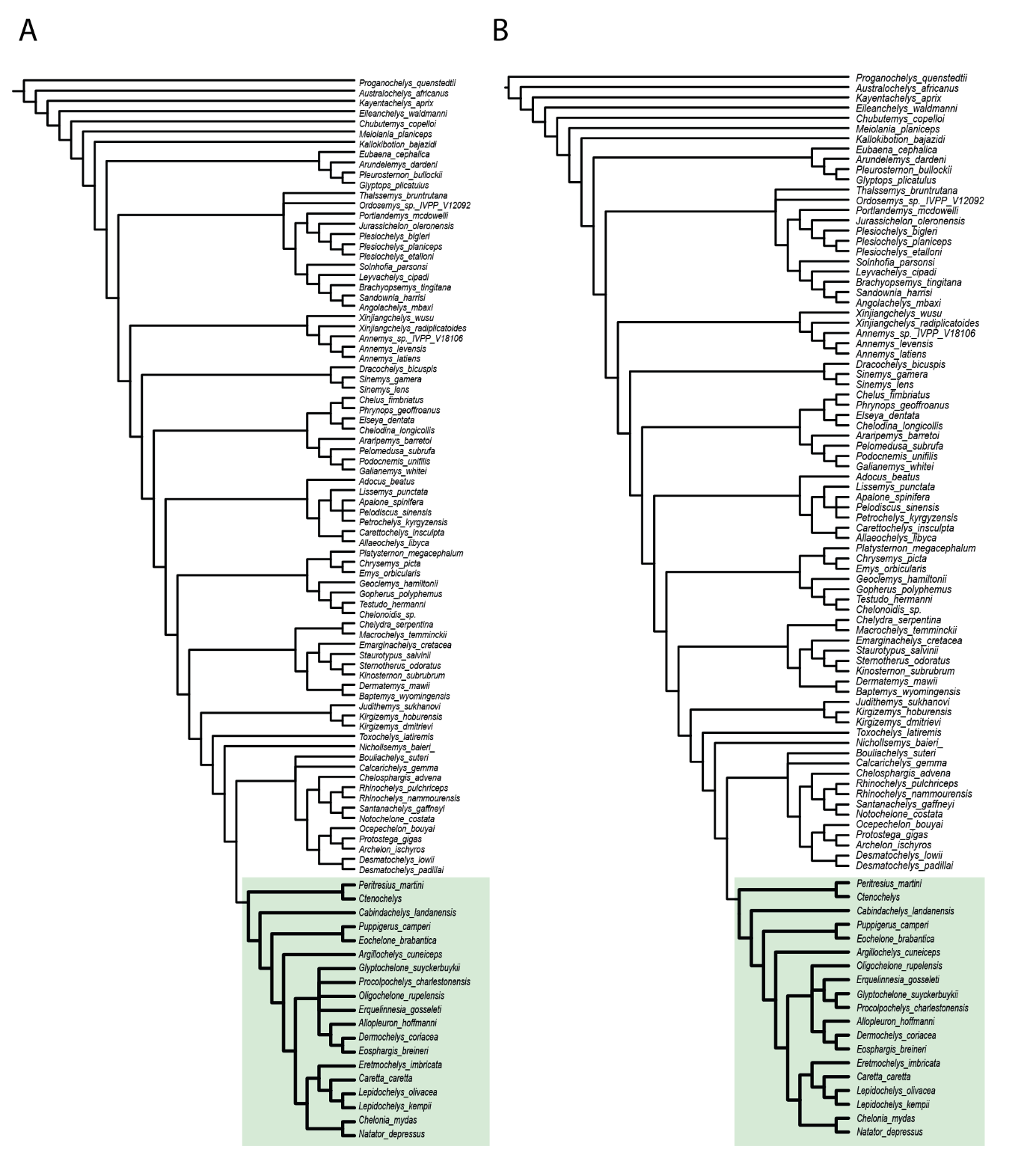
**

**Figure.7** Comparisons with strict consensus trees with or without additions of the new characters (ch.201, ch.206 and ch.207). A. Strict consensus tree performed with implied weighting (k=12) with addition of the new characters. B. Strict consensus tree performed with implied weighting (k=12) with new characters disabled.
