# Supplementary material for: A redescription of Glyptochelone suyckerbuykii (Ubaghs, 1879), an enigmatic fossil sea turtle (Chelonioidea) from the Maastrichtian of the Netherlands and Belgium, sheds new light on fossil sea turtle shell variation and neural bone homology
Source: Swiss J Palaeontol. 2025 Oct 1;144(1):62. doi: 10.1186/s13358-025-00389-y (PMC12488835; doi:10.1186/s13358-025-00389-y)
Supplement: Supplementary file 9 — Additional file 9. Description of neural series of Chelonia mydas. [file 13358_2025_389_MOESM9_ESM.docx]

**Description of the median shell elements of SMF 63250 (Fig. 16):**

The specimen used here for comparison, SMF 63250, consist in a complete juvenile skeleton that was scanned by X-ray tomography (see methods), the CT scan slides are available in Morphosource (https://www.morphosource.org/concern/media/000655871?locale=en) as well as the newly segmented models (https://www.morphosource.org/concern/media/000655871). The total length of the shell is about 33 cm long. We will only describe here the medial shell elements of SMF 63250 because we do not use the rest of the specimen.

**Nuchal**

The nuchal of SMF 63250 contacts anterolaterally the first peripherals; posterolaterally the first costals and posteromedially the preneural. The nuchal of SMF 63250 is roughly rectangular, wider than long and present lateral curved rims that form the medial border of the first costoperipheral fontanelles. The anterior rim of the nuchal of SMF 63250 present a shallow emargination and its visceral face displays an elevated nuchal pedestal for the accommodation of the eight cervical vertebrae.

**Preneural**

The preneural of SMF 63250 contacts the nuchal anteriorly, the first costals laterally, the first neural posteriorly and the thoracic neural arch I ventrally. The shape of the preneural of SMF 63250 is roughly quadratic with a slightly convex anterior rim and a slightly concave posterior rim. Its visceral face presents a median ridge at is central third that is anteroposteriorly short and dorsoventrally hight. This ridge inserts the preneural within a groove present in first thoracic neural arch dorsal most end.

**Neural series**

The neural one of SMF 63250 contacts the preneural anteriorly, the costal one anterolaterally, the costal 2 posterolaterally, the neural 2 posteriorly, the centrum of thoracic vertebrae I anteroventrally and the thoracic centrum II posteroventrally. The neural I of SMF 63250 is small, rectangular and longer than wide. Combined with the preneural, the neural I of SMF 63250 forms a 6A shape (Pritchard, 1988). The neural I of SMF 63250, as the subsquents neurals II to VIII, displays a fusion with the neural arch of their corresponding underlying thoracic vertebrae. The neurals II to VIII are longer than wide and displays all a 6A shape although they become significatively shorter toward the posterior end of the series. The neurals II to VIII of SMF 63250 displays respectively anterior contacts with the neural I to VII, anterolateral contacts with the costals I toVII; posterolateral contacts with the costals II to VIII, anteroventral contact with the thoracic centra II to VIII and posteroventral contacts with the thoracic centra III to IX. The neurals II to VII displays respectively posterior contacts with the neurals III to VIII whereas the neural VIII share its posterior margin with the postneural. The neural arches fused with the neural of SMF 63250 are stout and displays clear ovoid faced for the rib heads articulations. Moreover, these neural arches are anteriorly and posteriorly prolonged by median ridge itself carved at its midline by the neural canal. These median ridges are sutured with their anterior and posterior neighbors at the visceral side of inter-neurals sutures obliterating partially the aperture between the visceral face of the inter-neural sutures and the thoracic centra.

**Postneural**

The postneural of SMF 63250 contacts the neural VIII anteriorly, the costal VIII laretally, the suprapygal I posteriorly and the thoracic neural arch X ventrally. The postneural of SMF 63250 is smaller than the eight neural, long as wide and nearly rounded. The visceral face of the postneural of SMF63250 displays a low ridge median at its anterior half that insert within the dorsalmost aspect of the tenth thoracic vertebrae.

**Thoracic centra and free neural arches**

The first neural arch of SMF 63250 contacts the preneural dorsally, the eight cervical vertebra anteroventrally; the thoracic centrum 1 posteroventrally and the first neural posterodorsally. This neural arch is short and hight and displays obliquely ventrally oriented prezygapophyses that are prolonged ventrally by two small facets completing the articulation area for the eight cervical presents in the thoracic centrum I. The lateral processes of the first neural arch are short and flat and forms the anterior half of the first rib articulation facet whereas the neural spine process is high rounded and carved by a median groove accommodating with the median ventral ridge of the preneural. The thoracic centrum I contacts the neural arch I anteriorly, the neural I dorsally and the thoracic centrum II posteriorly. The thoracic centrum I is procoelous, longer than wide, although it is short. Its anterior facet that articulates with the eight thoracic vertebrae is anteroventrally directed. The dorsal surface of the first centra is deeply carved by the median neural canal whereas its ventral surface displays a distinct median ridge. The following centra II to IX displays a platycoelous morphology, they are longer than wide although they become shorter toward the posterior end of the series. They contact respectively the thoracic centra I to VIII anteriorly, the neurals I to VIII anterodorsally, the rib head II to IX anterolaterally, the rib head III to X posterolaterally, the neurals II to VII and the neural arch X posterodorsally and the thoracic centra III to X posteriorly. The thoracic centra II to VIII of SMF 63250 displays all a typical sandglass shape in ventral view due to the presence of a mediolateral constriction of their center and a dorsal face that is deeply incised by a deep and wide neural canal. A shallow and wide median ridge is perceptible on the ventral surface of the second thoracic centrum, but this structure disappears more posteriorly. The tenth neural arch of SMF 63250 contacts the neural VIII anteriorly, the postneural anterodorsally, the syprapygal I posterodorsally, the costals 10 laterally, the thoracic centrum X posteroventrally and the thoracic centrum IX ventrally. The neural spine process is distinct, elongated and rounded, a median shallow groove for the accommodation with the visceral side of the postneural is barely perceptible at its dorsal aspect. The ventral wall of the thoracic neural arch X is deeply carved by the neural canal. The tenth thoracic centrum of SMF 63250 is smaller and shorter than the precedent ones, displays a procoelous morphology. It contacts the nineth thoracic centrum anteroventrally, the thoracic neural arch X anterodorsally, the tenth rib anterolaterally and the first sacral vertebrae posteriorly. The dorsal face of the tenth thoracic centrum of SMF 63250 is carved by the neural canal that becomes narrower on this bone. The posterior facet of the tenth thoracic centrum of SMF 63250 is large and anteroposteriorly convex, it becomes more pointed ventrally.

**Suprapygals**

SMF 63250 displays two suprapygals. The suprapygal I contacts the postneural anterodorsally, the tenth thoracic neural anteroventrally and the suprapygal II posteriorly. The suprapygal I of SMF 63250 is strongly wider than long and displays a nearly anteriorly directed triangular shape with concave anterior and posterior rims. Its visceral surface is nearly flat, nevertheless it displays an anterior, low but distinct short and smooth median ridge that is situated slightly posterior to its contact with the tenth thoracic neural arch.

The second suprapygal of SMF 63250 contacts the suprapygal one anteriorly and the pygal posteriorly, it is notably longer than wide and displays a roughly posteriorly directed triangular shape.

**Pygal**

The pygal of SMF 63250 contacts the suprapygal II anteriorly and the eleventh peripherals laterally. The pygal of SMF 63250 displays an slightly wider than long trapezoidal shape and its dorsoventral cross section is strongly flattened and ovoid.
